# Humoral immune responses against gut bacteria in dogs with inflammatory bowel disease

**DOI:** 10.1371/journal.pone.0220522

**Published:** 2019-08-01

**Authors:** Sirikul Soontararak, Lyndah Chow, Valerie Johnson, Jonathan Coy, Craig Webb, Sara Wennogle, Steven Dow

**Affiliations:** Department of Clinical Sciences, College of Veterinary Medicine and Biomedical Sciences, Colorado State University, Fort Collins, Colorado, United States of America; University of California Los Angeles, UNITED STATES

## Abstract

Inflammatory bowel disease (IBD) in dogs is associated with clinical signs of intestinal dysfunction, as well as abnormal lymphocytic and myeloid cell infiltrates in the small and/or large intestine. Thus, in many respects IBD in dogs resembles IBD in humans. However, the factors that trigger intestinal inflammation in dogs with IBD are not well understood and have been variously attributed to immune responses against dietary antigens or intestinal antigens. Previous studies in humans with IBD have documented increased production of IgG and IgA antibodies specific to intestinal bacteria, and this abnormal immune response has been linked to disease pathogenesis. Therefore, we investigated the humoral immune response against gut bacteria in dogs with IBD, using flow cytometry to quantitate IgG and IgA binding. Studies were also done to investigate the source of these antibodies (locally produced versus systemic production) and whether greater antibody binding to bacteria is associated with increased inflammatory responses. We found that dogs with IBD had significantly higher percentages and overall amounts of IgG bound to their intestinal bacteria compared to healthy dogs. Similarly, significantly higher percentages of bacteria were IgA^+^ bacteria were also found in dogs with IBD. Serum antibody recognition of gut bacteria was not different between healthy dogs and dogs with IBD, suggesting that anti-bacterial antibodies were primarily produced locally in the gut rather than systemically. Importantly, bacteria in the *Actinobacteria* phylum and in particular the genus *Collinsella* had significantly greater levels of antibody binding in dogs with IBD. Based on these findings, we concluded that antibody binding to commensal gut bacteria was significantly increased in dogs with IBD, that particular phyla were preferential targets for gut antibodies, and that anti-bacterial antibody responses may play an important role in regulating gut inflammation.

## Introduction

Inflammatory bowel disease (IBD) in dogs is characterized by infiltration of lymphocytes and macrophages into the mucosa and submucosa and clinical signs of GI dysfunction (diarrhea, malabsorption, weight loss) [1, 2]. It has been proposed that alteration of the gut environment and development of dysbiosis may allow the overgrowth of pathogenic bacteria and induction of intestinal injury and inflammation in IBD [3]. Genetic and environmental factors are also associated with IBD in dogs and humans [4, 5]. Inflammation in IBD in humans is thought to be mediated by both cellular and humoral immune mechanisms [6–8]. Increasingly, studies in IBD in humans (e.g., Crohn’s disease, ulcerative colitis) have focused on the role of immune responses targeted to gut bacteria, as opposed to immune responses targeting gut tissues or dietary antigens [9–12].

In dogs with IBD, previous studies have documented dysregulation of humoral immunity, principally a reduction in the overall amount of gut IgA production [13]. For example, decreased production of mucosal IgA has been documented in dogs with IBD, along with increased production of pro-inflammatory cytokines by gut mucosal immune cells (T cells and macrophages) [13–15]. However, the specificity of gut IgA in dogs has not been previously investigated. Increased numbers of plasma cells have also been observed in the lamina propria of dogs with IBD, consistent with local IgG production [16, 17].

Studies in humans with IBD have found significantly increased numbers of fecal bacteria with bound IgG, especially in patients with Crohn’s disease (CD) and ulcerative colitis (UC) [12, 18, 19]. It has also been shown in some cases that IBD patients have greater numbers of IgA^+^ bacteria [12, 20]. Interestingly, studies found that the IgG and IgA antibodies preferentially bound to certain pathogenic bacteria in CD patients, including *Clostridium coccoides*, *E*. *coli*, and *Pseudomonas fluorescens* [21–23].

Little is known however regarding antibody recognition of gut bacteria in dogs with IBD. It is known that the microbiome is very different in dogs with IBD than in healthy dogs and that certain phyla predominate in these bacterial populations [24, 25]. For example, a dysbiosis index has been created by AlShawaqfeh *et al*. to measure the degree to which the normal flora has been disrupted by an overgrowth of bacteria associated with clinical signs in dogs with IBD [26].

Therefore, in the present study, we investigated humoral immune responses to gut bacteria in dogs with IBD and compared these responses to those present in healthy dogs. To address this question, the amounts of IgG and IgA antibodies bound to the surface of fecal bacteria was assessed using flow cytometry. The presence of circulating anti-bacterial antibodies in blood was also evaluated. To determine whether bacterial antibodies in dogs with IBD were specific for certain bacteria, fecal bacteria with high levels of surface IgG were flow sorted and subjected to 16S rRNA sequencing. Finally, we also evaluated the impact of IgG binding to gut bacteria on the host innate immune response and macrophage activation, using in vitro assays. The studies reported here provide important new insights into the pathogenesis of IBD in dogs and suggest that local humoral immune response against gut bacteria play an important role in disease pathogenesis. Moreover, these studies further illustrate the potential value of the dog spontaneous IBD model for investigating new strategies for immunotherapy and microbiome modulation.

## Material and methods

### Study populations

A prospective observational study was conducted at the Colorado State University Veterinary Teaching Hospital (CSU-VTH). All animal studies were approved by the Institutional Animal Care and Use Committee (IACUC) and the Clinical Review Board (CRB) at CSU (#VCS 2016–084). Dog owners were informed regarding the study protocol, and consent was obtained before enrollment in the study. A total of 29 dogs, 20 dogs diagnosed with IBD, and 9 healthy control dogs, were evaluated in the study.

#### IBD dogs

Twenty dogs with IBD (14 males and 6 females) with persistent signs of gastroenteritis, including vomiting, diarrhea, weight loss, for a minimum of 3 weeks were recruited into the study. All study dogs with IBD had undergone endoscopy and intestinal biopsy to confirm a diagnosis of IBD and rule out intestinal lymphoma. Most animals had previously undergone and failed food trials including elimination diet, novel protein and/or hydrolyzed protein at least 3 weeks. All dogs had no recent history of receiving immunosuppressive medications and were free from other diseases causing chronic GI dysfunction, including hepatic disease, pancreatic insufficiency, metabolic disease parasitic disease, and renal disease. The study purposely excluded German Shepherd dogs, as this breed is known to be predisposed to defective intestinal IgG and IgA production [27, 28].

All study dogs with IBD received clinical evaluation including disease activity index evaluation (Canine Inflammatory Bowel Disease Activity Index; CIBDAI [29] and Canine Chronic Enteropathy Clinical Activity Index; CCECAI [30]), CBC, serum chemistry profile, and fecal examination for parasites. The H&E stained intestinal biopsy specimens from dogs with IBD underwent WSAVA histopathologic score evaluation [1, 31] by a board-certified veterinary pathologist. Additional tests performed in dogs with IBD included serum folate concentration and serum cobalamin concentration (Gastrointestinal Laboratory, Texas A&M University, TX).

#### Clinical healthy controls animals

Nine clinically healthy dogs (4 males and 5 females) that were also age-matched to the IBD dogs were enrolled in the study. These dogs were client owned and were evaluated at the CSU-VTH for a health checkup. Based on history and normal physical examination, these animals were judged to not have any clinical signs indicative of gastrointestinal disease, and the animals had no history of immune-mediated disease or immune suppressive medication usage. All dogs had complete blood count (CBC) and serum chemistry profiles performed, and all evaluations were within the normal limits.

### Sample collection

Stool samples and blood samples from all study dogs were collected and stored at 4°C immediately prior to sample preparation, which occurred within 4 hours of sample collection. Serum samples were stored at -80°C. Stool samples were obtained by spontaneous defecation and/or rectal palpation. Fresh stool samples were processed to generate a fecal bacteria suspension as described previously [18]. Briefly, 0.5g of stool was homogenized in 24.5 ml of sterile-filtered phosphate buffer saline (PBS; 0.2μm-filtered) solution using vortexing, then centrifuged at 700 x G for 5 minutes. The washed bacteria were collected and stored in 1 ml aliquots at -80°C until used.

### Flow cytometry

The fecal bacteria suspension was centrifuged at 10,000 x G for 5 minutes to obtain a bacterial pellet, which was washed with PBS once. For measurement of Ig-binding fecal bacteria, the bacterial pellet was resuspended in 100μl of either rabbit anti-dog IgG-Alexa Fluor 647 conjugate (Jackson ImmunoResearch Laboratories, PA, USA; diluted 1:200 in PBS plus 1% BSA, or with a solution of goat anti-dog IgA-FITC conjugate (Lifespan Biosciences, MA, USA, also diluted 1:200 in PBS plus 1% BSA, and incubated for 30 minutes on ice. The suspensions were then washed twice and then fixed for 10 minutes in a solution of 4% paraformaldehyde (PFA). After washing, the bacteria were resuspended in 380 μl of PBS, plus 20 μl propidium iodine solution (PI; 1 g/ml; Sigma-Aldrich, St. Louis, MO, USA), which was added to each sample before flow cytometry analysis.

For detection of serum IgG antibody specific to intestinal bacteria, 6 stock cultures of *Escherichia coli* (*E*. *coli*) isolated from the stool of healthy dogs (n = 3) and dogs with IBD (n = 3) were generated as described in 2.4. The bacteria in short term cultures were collected for detection of IgG binding, using serum from healthy dogs and dogs with IBD followed the previous study method [32]. Briefly, each test serum sample was diluted 1:200 in PBS plus 1% BSA, then added to *E*. *coli* in suspension and incubated 30 minutes on ice, then washed followed by the rabbit anti-dog IgG-Alexa Fluor 647 conjugate, and analyzed by flow cytometry, as described previously.

Flow cytometric analysis of fecal samples for IgG and IgA binding was performed using a Beckman Coulter Gallios flow cytometer (Brea, CA, USA). Analysis was done on 100,000 PI-positive events (PI staining was done to include bacteria (DNA^+^) for analysis and exclude debris without nuclear material (DNA^-^) from analysis). Flow cytometry data were analyzed using FlowJo Software (Ashland, OR, USA). The analysis included the percentage of positive fluorescent cells as well as the fluorescence intensity of IgG^+^ or IgA^+^ cells. Background fluorescence levels were determined using bacteria without addition of anti-IgG or IgA antibodies. An example of the typical gating scheme is provided in S1 Fig.

### Isolation of *E*. *coli* intestinal strains and evaluation of anti-bacterial antibodies present in serum

Six different isolates of *E*. *coli*, 3 obtained from feces of healthy dogs and 3 from dogs with IBD, were prepared to assess the presence of anti-bacterial antibodies in serum of healthy dogs and dogs with IBD. Though we acknowledge that many different species of bacteria were recognized by antibodies using fecal flow cytometry (see below), we reasoned that *E*. *coli* could be readily propagated in pure culture from the gut and might be a useful proxy for enteric bacteria in general. Also, the assays were done using pure cultures of *E*. *coli* obtained from the GI tract of dogs to avoid the confounding effects of IgG already present on the surface of GI bacteria obtained directly from feces. In addition, isolates were obtained from healthy dogs and dogs with IBD in case the strains differed based on dog disease status.

To isolate *E*. *coli*, fresh fecal samples of IBD dogs and healthy dogs were collected, homogenized, and diluted in PBS. The fecal suspension was cultured in Tryptic Soy Broth (TSB) (BD, Franklin Lake, NJ, USA) at 37°C overnight with shaking. The overnight cultured media was plated on McConkey agar and incubated in aerobic condition overnight at 37°C. The cultured colonies were examined the next day, and each *E*. *coli*-suspected colony was further subcultured onto blood agar as well as McConkey agar in parallel. The next day, the pure cultures were submitted to confirm the *E*. *coli* species by evaluation at the CSU-VTH diagnostic lab.

Six *E*. *coli* isolates (3 from dogs with IBD and 3 from normal dogs) were used to test for the relative concentrations of anti-bacterial IgG antibodies present in the serum of dogs with IBD and healthy dogs. Briefly, each pure *E*. *coli* isolate were cultured in aerobic condition overnight at 37°C with shaking. The pure *E*. *coli* cultured suspension was washed with PBS and centrifuged to get a bacterial pellet. The *E*. *coli* pellet was resuspended in 100 μl of dog serum dilution and followed the staining protocol as previously described. Briefly, diluted dog serum was incubated with *E*. *coli* on ice for 30 minutes, the bacteria were washed twice, and then incubated with anti-dog IgG or IgA secondary antibody for 30 minutes. The bacterial pellets were washed, fixed with 4% PFA and PI was added before flow cytometry analysis.

### Macrophage isolation and culture

Macrophages were derived from differentiated monocytes from blood of healthy dogs as described previously [33], and were used to assess macrophage activation following incubation with fecal bacteria recovered from healthy dogs and dogs with IBD. Briefly, PBMC were isolated from EDTA-anticoagulated blood samples by Ficoll-density separation, and the PBMC were resuspended in complete medium (DMEM, 1% Penicillin-streptomycin, essential and non-essential amino acid) with 1% FBS and plated at a density 1x10^6^ PBMC/0.5 ml in 48-well polystyrene cell culture plates, incubated for 4 hours at 37°C. After allowing for monocyte adhesion, the non-adherent cells were washed off with PBS and the remaining monocytes were refed with complete medium with 15% FBS, supplemented with 10 ng/ml huM-CSF (Peprotech, Rocky Hill, NJ, USA) and cultured for 7 days. The medium was changed every 2 days and after 7 days in culture, the monocyte-derived macrophages were used for phagocytosis and cytokine assays.

### Macrophage phagocytosis and activation assays

Fecal bacteria (prepared as noted above) from dogs with active IBD (n = 5), and normal dogs (n = 5) were used in macrophage phagocytosis and activation assays. To assess bacterial phagocytosis, numbers of bacteria (note that bacteria used in these assays were non-viable after freezing) added to macrophage cultures were calculated and equalized by first determining bacterial counts using PI-labeled bacteria and calibrating counts using counting beads (Invitrogen, Eugene, OR). Final numbers of bacteria in samples were calculated by comparing the ratio of bead events to bacterial cell events according to the manufacturer datasheet. The fecal bacteria were added to macrophages at MOI ratio of 5 bacteria per 1 macrophage, and bacteria were spun onto macrophages by centrifugation at 2000 x G for 10 minutes, then the macrophages were incubated for 2 hours at 37°C. The cultures were then washed to remove non-phagocytosed bacteria and the cells were detached and performed the flow cytometry. The % of PI+ve macrophage and PI abundance in macrophage were analyzed.

To assess macrophage activation by fecal bacteria, macrophages were incubated with bacteria (MOI = 5) for 2 hours to allow phagocytosis, then the non-phagocytosed bacteria were removed, and the macrophages cultured for an additional 24 hours. The supernatants were collected to measure cytokines (TNF-α, IL-10) by ELISA. As a positive control for cytokine release and activation, 10 ng/ml LPS was added to parallel cultures of macrophages. These assays were repeated 3 times using blood from 3 different unrelated donor animals to assure reproducibility.

### Flow sorting and 16S rRNA sequencing

For these studies, 3 populations of bacteria were analyzed for population composition, using 16S sequencing. The 3 populations consisted of total fecal bacteria from dogs with IBD (n = 10), fecal bacteria from healthy dogs (n = 10), and bacteria with high levels of bound IgG (IgG^hi^ bacteria), obtained from feces of dogs with IBD (n = 10) following incubation with anti-dog IgG secondary antibody, and prepared by cell sorting using a BD FACSAria sorter. To enrich for IgG^+^ bacteria, fecal bacteria from dogs with IBD were immunostained as noted above, and the population of IgG^hi^ bacteria (MFI greater than normal baseline) was sorted. The reference population for setting sorting gates was comprised of unstained bacteria. The purity of the sorted bacterial population was assessed by flow cytometry and was found to consist of at least 85% IgG^hi^ bacteria.

Bacteria were subjected to 16S rRNA sequencing following DNA extraction using a Mobio PowerSoil DNA Isolation kit (Qiagen, Valencia, CA) according to manufacturer’s instructions. Extracted DNA was submitted for 16S rRNA sequencing and analyzed by Novogene Corporation (Chula Vista, CA). The 16S rRNA sequencing was performed as reported in a previous study [34]. Sequences analysis were performed by Uparse software (Uparse v7.0.1001 http://drive5.com/uparse/) [35]. For each representative sequence, Mothur software was performed against the SSUrRNA database of SILVA Database (http://www.arb-silva.de/) [36]. For species annotation at each taxonomic rank (Threshold:0.8~1), OTUs abundance information was normalized using a standard of sequence number corresponding to the sample with the least sequences. Subsequent analysis of alpha diversity and beta diversity were all performed basing on this output normalized data. Alpha diversity was calculated using Shannon diversity index. Beta diversity on both weighted and unweighted unifrac were calculated by QIIME software (Version 1.7.0). PCoA analysis was displayed by WGCNA package, stat packages and ggplot2 package in R software (Version 2.15.3). Metastats was calculated by R software. P-values were calculated by the method of permutation test while q-values were calculated by method of Benjamini and Hochberg False Discovery Rate [37]. Anosim, MRPP and Adonis were performed by R software (Vegan package: anosim function, mrpp function and adonis function). AMOVA was calculated by mothur using amova function. T-test and drawing were conducted by R software.

### Statistical analysis

Data were analyzed using Prism 7 software (GraphPad, San Diego, CA, USA). The normality of data was initially analyzed using the Shapiro-Wilk normality test. The normally distributed data were shown as mean ± standard deviation (SD). Data which were not normally distributed were reported in median (range). Statistical differences between 2 groups were evaluated using the unpaired t-test for parametric data and Mann-Whitney test for non-parametric data as indicated in the text. For statistical assessment of serum IgG response, the % IgG binding *E*. *coli* were compared using one-way ANOVA. The result from repeated experiments including cytokine production from different PBMC donors was normalized to baseline control before analysis. To analyze the association between Ig-binding bacteria and other variables including disease activity index, linear regression analysis was performed. The Receiver-Operating Characteristic (ROC) curve was used to determine the sensitivity and specificity as a diagnostic ability between IBD and normal dogs. In all studies, the statistical significance was set at *P* < 0.050. Dataset generated and analyzed during the current study including 16s rRNA sequencing are available in an open access repository (https://doi.org/10.5061/dryad.90qg722).

## Results

### Breed characteristics of study dogs

The demographic data and disease activity index evaluation of 20 dogs with IBD enrolled in the study were shown in the Table 1. The breeds included Bernese Mountain Dog (n = 4), mixed breeds (n = 4), Labrador Retriever (n = 2), Yorkshire Terrier (n = 2), Pug, Rottweiler, Boxer, Cavalier King Charles Spaniel, German Shorthaired Pointer, English Bulldog, American Eskimo, Siberian Husky. The breeds in the healthy control group (n = 9) included mixed breed animals, Standard Poodle, Cocker Spaniel, Shih Tsu, Nova Scotia Duck Tolling Retriever, English Coonhound, Chihuahua, English Setter. On average, the disease duration in dogs with IBD was classified as chronic, with moderate disease activity index (S1 Table). Summary of histopathologic findings in IBD group was reported as following; briefly, eleven of dogs with IBD had moderate lympho-plasmacytic inflammation of the duodenum, in five cases associated with mixed eosinophilic/neutrophilic infiltration. Six dogs had mild lympho-plasmacytic inflammation and a half of them were found to have mixed eosinophilic infiltration. Three cases were documented with severe lympho-plasmacytic inflammation and two were mixed infiltrates. Additional observations included glandular degeneration, crypt abscesses, lacteal dilatation, villous fusion and villous shortening. Endoscopic scores, histopathologic findings and WSAVA histopathology scores were also reported in Table 1 and S1 Table. There was no significant difference in age between healthy control dogs and dogs with IBD (*P* = 0.42). However, the average body condition score in healthy dogs was greater than IBD dog (*P* = 0.01), which is a common finding since the IBD animals typically exhibit weight loss. The diet consumed by study dogs during the period of study was also shown in S1 and S2 Tables as it is well recognized that diet can influence microbial populations and metabolomic profiles.

**Table 1:** Demographic data of study groups.

	IBD	Normal
**Sample size**	20	9
**Gender**		
**-Male**	14	4
**-Female**	6	5
**Age (year)**	6.4 ± 3.77	7.6 ± 3.05
**Weight (kg)**	24.04 ± 13.79	22.73 ± 11.35
**BCS (9 scales)**	4.1 ± 1.13	5.33 ± 0.86
**Disease Duration (month)**	4.62 ± 5.27	-
**Disease activity index**		NP
**-CIBDAI**	6.7 ± 3.57	
**-CCECAI**	8.1 ± 4.55	
**Endoscopic lesion**	100%	NP
**Endoscopic score**		NP
**-Gastroscopic**	1 (0–2)	
**-Duodenoscopic**	3 (2–6)	
**-Ileoscopic**	3 (1–7)	
**WSAVA Histopathology score**	5.15 ± 2.68	NP

Data reported as Mean ± SD and Median (range).

CIBDAI = Canine Inflammatory Bowel Disease Activity Index; CCECAI = Canine Chronic Enteropathy Clinical Activity Index; NP = not performed.

### IgG binding to fecal bacteria in dogs with IBD versus healthy dogs

**Fig 1:**
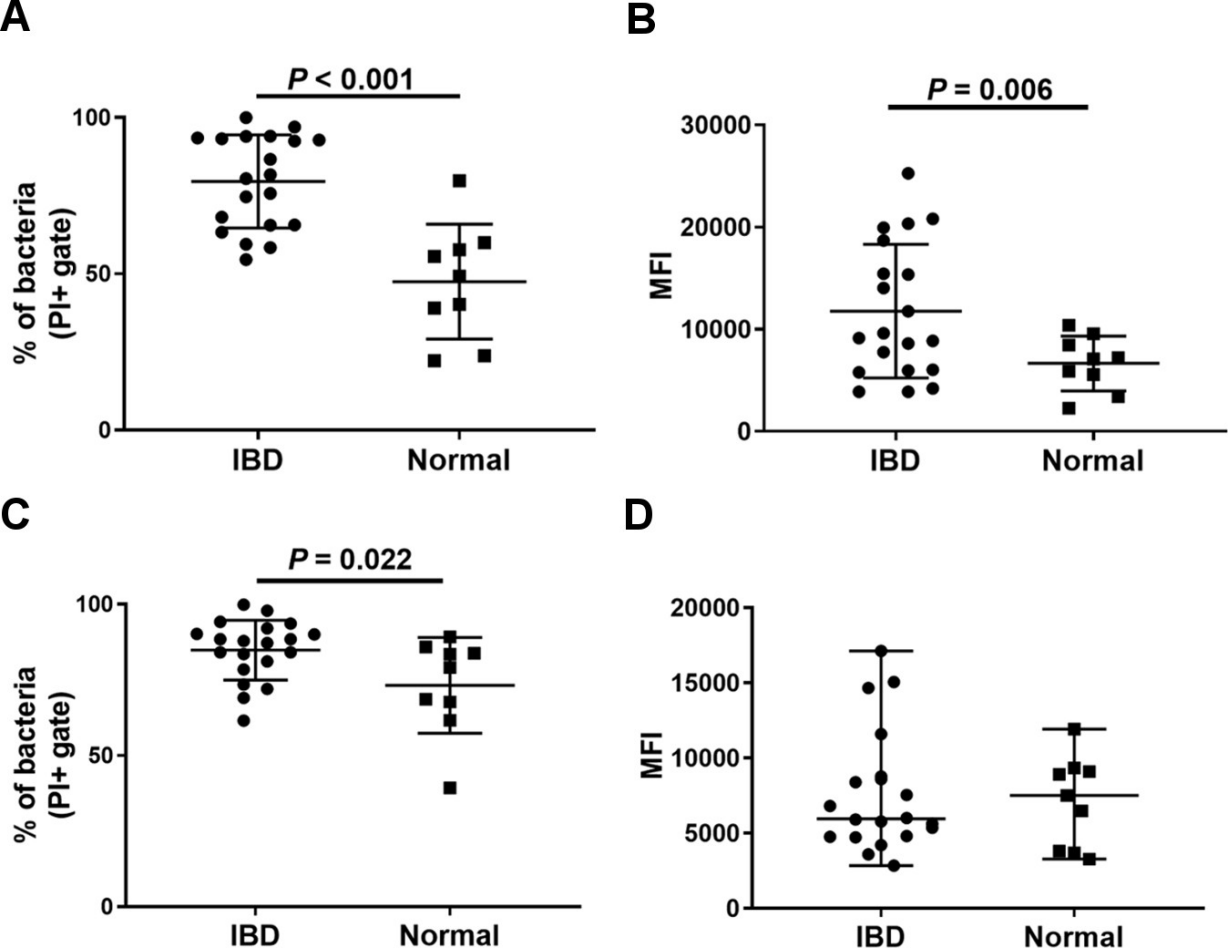
IgG^+^ and IgA^+^ fecal bacteria in healthy dogs and dogs with IBD. (A) The percentages of IgG^+^ bacteria are plotted in dogs with IBD versus healthy dogs. (B) The amount of IgG bound to each bacterium (MFI) is plotted for the two groups of animals. IgA binding percentages and total IgA binding to each bacterium are depicted in C and D, respectively. Data are plotted as Mean ± SD. Statistical differences were calculated using two-tailed unpaired t-test (A,B,C) or a Mann-Whitney U test (D).

The percentage of IgG^+^ bacteria in the feces of dogs with IBD was significantly greater than in feces of healthy control animals: (IBD: 80% ± 15.05; healthy: 47.5% ± 18.35, *P* < 0.0001, (Fig 1A). In addition, the overall amount of IgG bound by bacteria, as assessed by mean fluorescence intensity (MFI), was significantly higher in dogs with IBD than in healthy dogs (MFI-IBD: 11,769 ± 6,539 a.u.; MFI-healthy: 6,650 ± 2,687 a.u., *P* = 0.005, Fig 1B).

We found that the percentage of IgA^+^ bacteria was also significantly higher in dogs with IBD than in healthy dogs, though the magnitude of the difference was less than for IgG binding (IBD: 84.86% ± 9.87; healthy: 73.18% ± 15.83, *P* = 0.022, Fig 1C). However, the total amount of IgA bound to bacteria was not significantly different for the two groups of dogs (MFI-IBD: 7,607 (2,834–17,120) a.u.; MFI-healthy: 7,113 (3,280–11,925) a.u., *P* = 0.91, Fig 1D).

**Fig 2:**
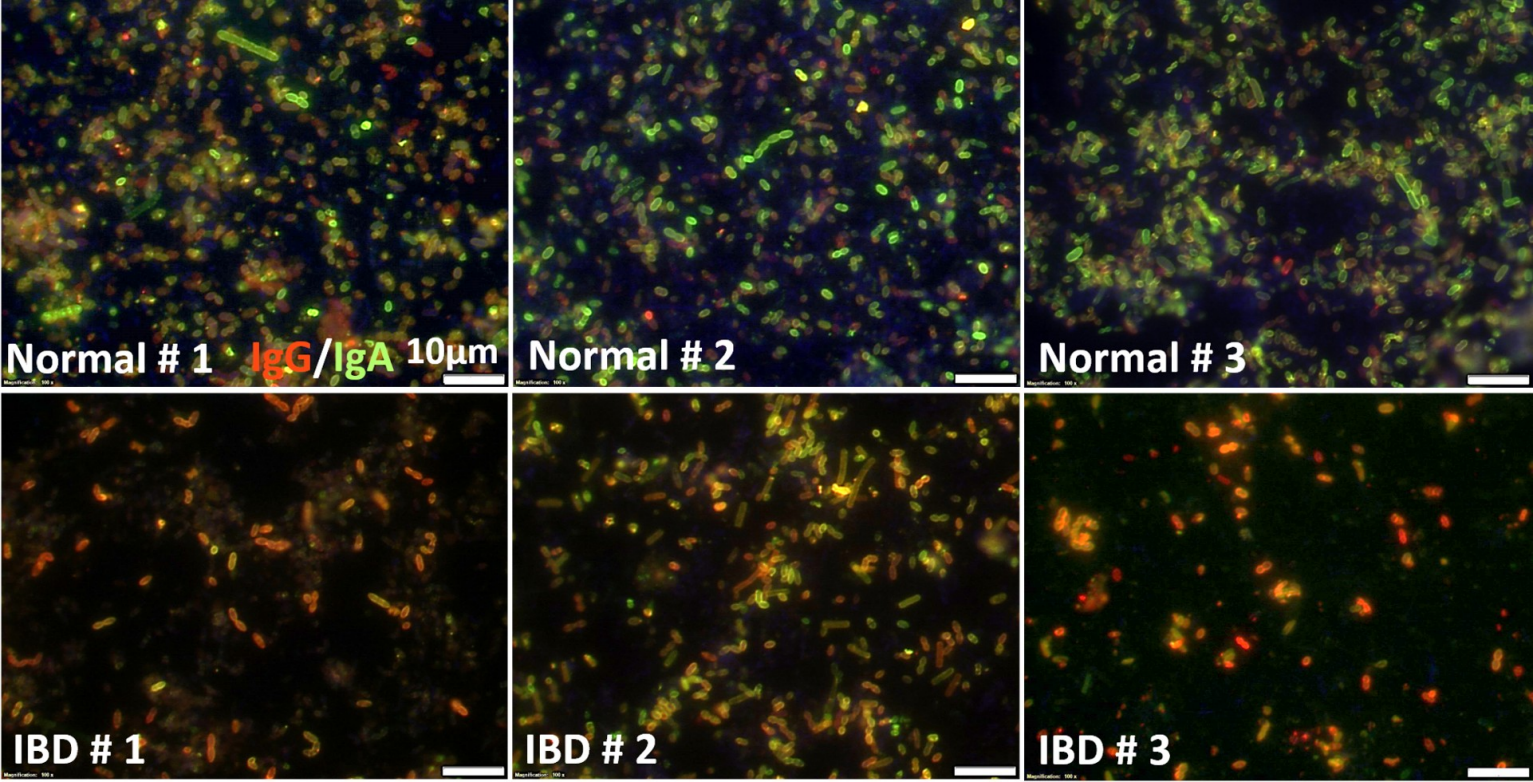
Ig-binding fecal bacteria. Immunofluorescence staining and imaging of fecal bacteria from a healthy dog (top row) and from a dog with IBD (bottom row). IgA bound to bacteria indicated as green, while IgG^+^ bacteria indicated as red. Bacteria with both bound antibodies show up as yellow images in merged figures. Scale bar indicates 10 μm.

Confocal microscopy was used to visualize the IgG^+^ and IgA^+^ populations of bacteria, and the potential overlap in the two populations of Ig^+^ bacteria, in dogs with IBD and healthy dogs (Fig 2). The IgG^+^ bacteria were visualized with rabbit anti-dog IgG-Cy3 conjugate (red) and the IgA^+^ bacteria were visualized with goat anti-dog IgA-FITC conjugate (green), and dual positive bacteria appeared yellow in merged images. In feces of healthy dogs, there was a predominant population of IgA^+^ bacteria, with substantially fewer IgG^+^ bacteria (Fig 2). In feces from dogs with IBD, many more IgG^+^ bacteria were present, as reflected by a large number of dual positive (yellow) bacteria visualized. Linear regression analysis revealed that there was significant correlation between the percentages of IgG^+^ and IgA^+^ bacteria in dogs with IBD (R^2^ = 0.45, *P* = 0.001; S2 Fig). A similar correlation of MFI of IgG^+^ and IgA^+^ bacteria was also noted (R^2^ = 0.48, *P* = 0.001), suggesting that increased in IgG binding activity was associated with increased IgA binding in terms of both percentages of bacteria bound and in the abundance of IgG and IgA present on the surface of bacteria. In the case of healthy dogs, there was not a significant correlation between IgG and IgA-binding bacteria.

### Recognition of fecal bacteria by circulating IgG

To determine whether the IgG bound to gut bacteria was produced primarily in the GI tract or was instead produced in extra-intestinal lymphoid tissues and then secondarily transported to the GI tract (e.g., by leakage from intestinal vasculature), we assayed serum from the presence of IgG antibodies specific for a common intestinal bacterium (*E*. *coli*) as described in Method.

**Fig 3:**
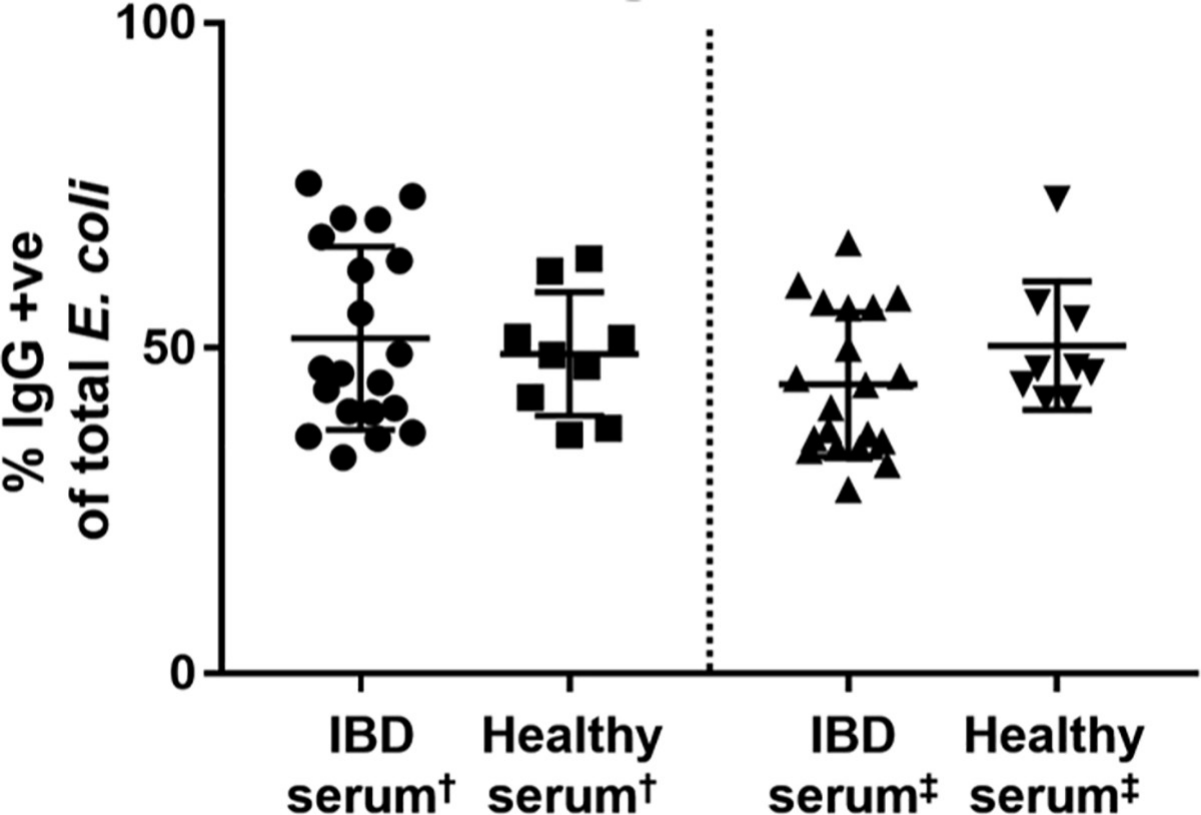
Serum IgG recognition of *E*. *coli* isolated from healthy dogs and dogs with IBD. Six separate fecal isolates of *E*. *coli* (3 from dogs with IBD and 3 from healthy dogs) were incubated with serum from dogs with IBD (n = 20) and healthy dogs (n = 9), and IgG binding to the surface of bacteria was quantitated using flow cytometry, as noted in Methods. Scatter plots depicting IgG^+^ bacteria percentages in healthy versus IBD dogs plotted. The percentages of IgG^+^ bacteria were not significantly different between the two groups of animal sera (*P* = 0.41). (†) Indicated *E*. *coli* isolates from normal dogs, while (‡) indicated *E*. *coli* isolates from dogs with IBD. Data were plotted as Mean ± SD. Statistical differences were calculated using one-way ANOVA.

We found that the amount of IgG present in serum that bound to *E*. *coli* was not different between healthy dogs and dogs with IBD (Fig 3). Nor were there differences in serum IgG recognition of *E*. *coli* isolated from healthy dogs or from dogs with IBD. Similarly, differences in serum IgA recognition of *E*. *coli* were not observed in healthy dogs versus dogs with IBD (S3 Fig). Thus, we concluded that the IgG bound to the surface of fecal bacteria was primarily produced locally in the GI tract, rather than being produced systemically. These findings are also consistent with the increased numbers of plasma cells detected in the GI tract of dogs with IBD, as described previously [16, 17].

### Macrophage phagocytosis of fecal bacteria increased in dogs with IBD

Given the presence of significantly more IgG^+^ bacteria in the GI tract of dogs with IBD, we next sought possible links between this phenomenon and induction of intestinal inflammation. One plausible mechanism linking IgG^+^ bacteria to GI inflammation could involve an interaction of gut bacteria with phagocytic cells such as macrophages. Therefore, we used an in vitro system to determine whether gut bacteria from dogs with IBD were inherently more inflammatory than gut bacteria from healthy dogs.

**Fig 4:**
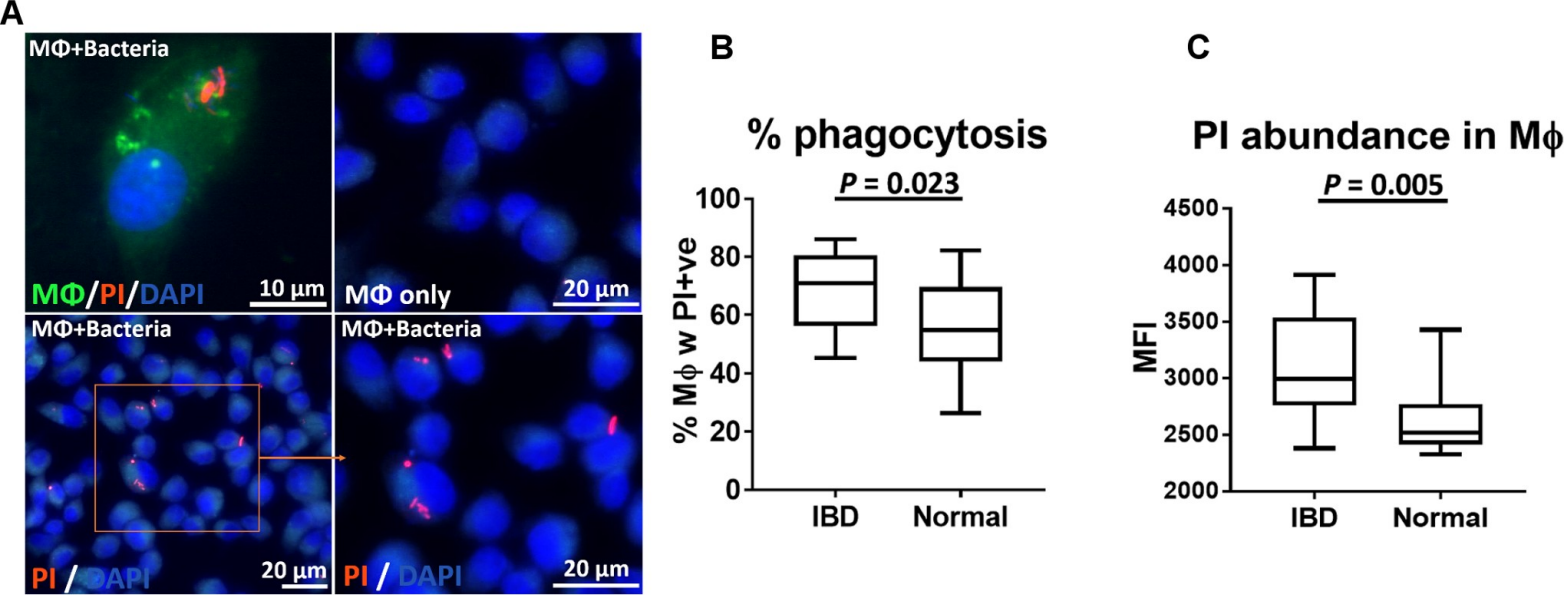
Macrophage phagocytosis of fecal bacteria from dogs with IBD versus healthy dogs. (A) Fecal bacteria (PI staining; red) from dogs with IBD and healthy dogs (n = 5 per group) were incubated with primary cultures of canine monocyte-derived macrophages and bacterial uptake was determined using flow cytometry, as described in Methods. Images were obtained using confocal microscopy, with PI stained bacteria visualized as red objects within cultured macrophages. DAPI staining (blue) demonstrates cell nuclei. Similar results were obtained in at least n = 3 repeated, independent studies. Box plot comparing the percentage of macrophages containing intracellular bacteria (B) and the relative number of bacteria per macrophage (C), when bacteria from dogs with IBD and healthy control dogs were compared. Statistical differences were calculated using unpaired t-tests (B) and by the Mann-Whitney test (C). Scale bar as indicated.

First, we compared the relative ability of dog macrophages to phagocytose bacteria from IBD dogs versus bacteria from healthy dogs (Fig 4). Macrophage phagocytosis of bacteria from IBD dogs was found to be significantly greater than phagocytosis of bacteria from healthy dogs. For example, the percentage of macrophages containing phagocytosed bacteria was 67.91 ± 13.68% in cultures incubated with bacteria obtained from IBD animals, compared to 55.05 ± 15.48% for bacteria from healthy dogs (*P* = 0.023, Fig 4B). Also, the average numbers of ingested bacteria per individual macrophage (as reflected by increased mean fluorescence intensity) was significantly increased in macrophages incubated with bacteria from dogs with IBD [MFI: 2,994 (2,378–3,912)] compared to macrophages fed bacteria from healthy dogs (MFI: 2,519 (2,323–3,428), *P* = 0.005, Fig 4C). Thus, GI bacteria in dogs with IBD were more likely to be phagocytosed by macrophages than bacteria from healthy dogs.

### Ingestion of bacteria from dogs with IBD triggers greater macrophage inflammatory response

**Fig 5:**
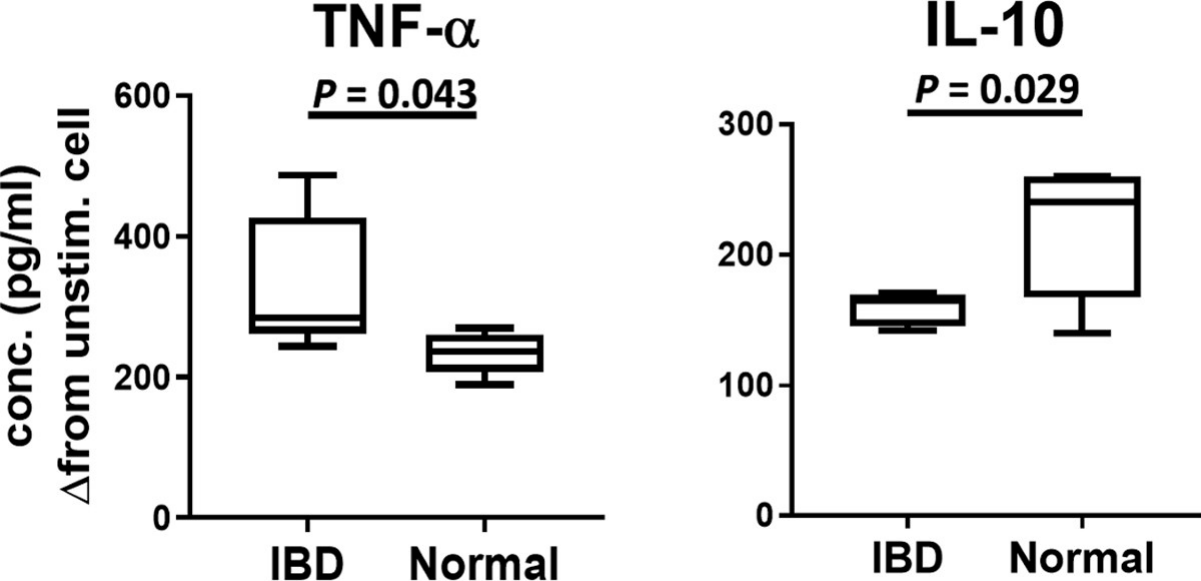
Cytokine production by activated macrophages. Canine monocyte-derived macrophages were activated by incubation and phagocytosis of non-viable fecal bacteria obtained from dogs with IBD (n = 5) and from healthy normal dogs (n = 5), as described in Methods. TNF-α and IL-10 concentrations in media obtained from macrophage cultures 24 hours after bacterial inoculation were measured using commercial canine-specific ELISA. Box plots comparing cytokine concentrations between the 2 groups of fecal bacterial samples are depicted. Statistical differences were calculated using unpaired t-tests. The assays were repeated for 3 times, total of 3 different PBMC donors.

Ingestion of bacteria, particularly via Fc receptor-mediated internalization, serves as a strong activating stimulus for macrophages [38]. Therefore, we next examined the impact of fecal bacteria ingestion on macrophage activation and cytokine production. We found that macrophages incubated with bacteria from IBD dogs produced significantly greater amounts of TNF-α than macrophages incubated with bacteria from healthy dogs (Fig 5). Conversely, macrophages incubated with IBD bacteria produced significantly less IL-10 than macrophages incubated with healthy dog bacteria (Fig 5). Thus, we concluded that bacteria present in the gut of dogs with IBD were inherently more immune stimulatory and capable of triggering macrophage production of pro-inflammatory cytokines such as TNF-α, than bacteria from the gut of healthy dogs.

### Microbiome analysis and selectivity of IgG binding

**Fig 6:**
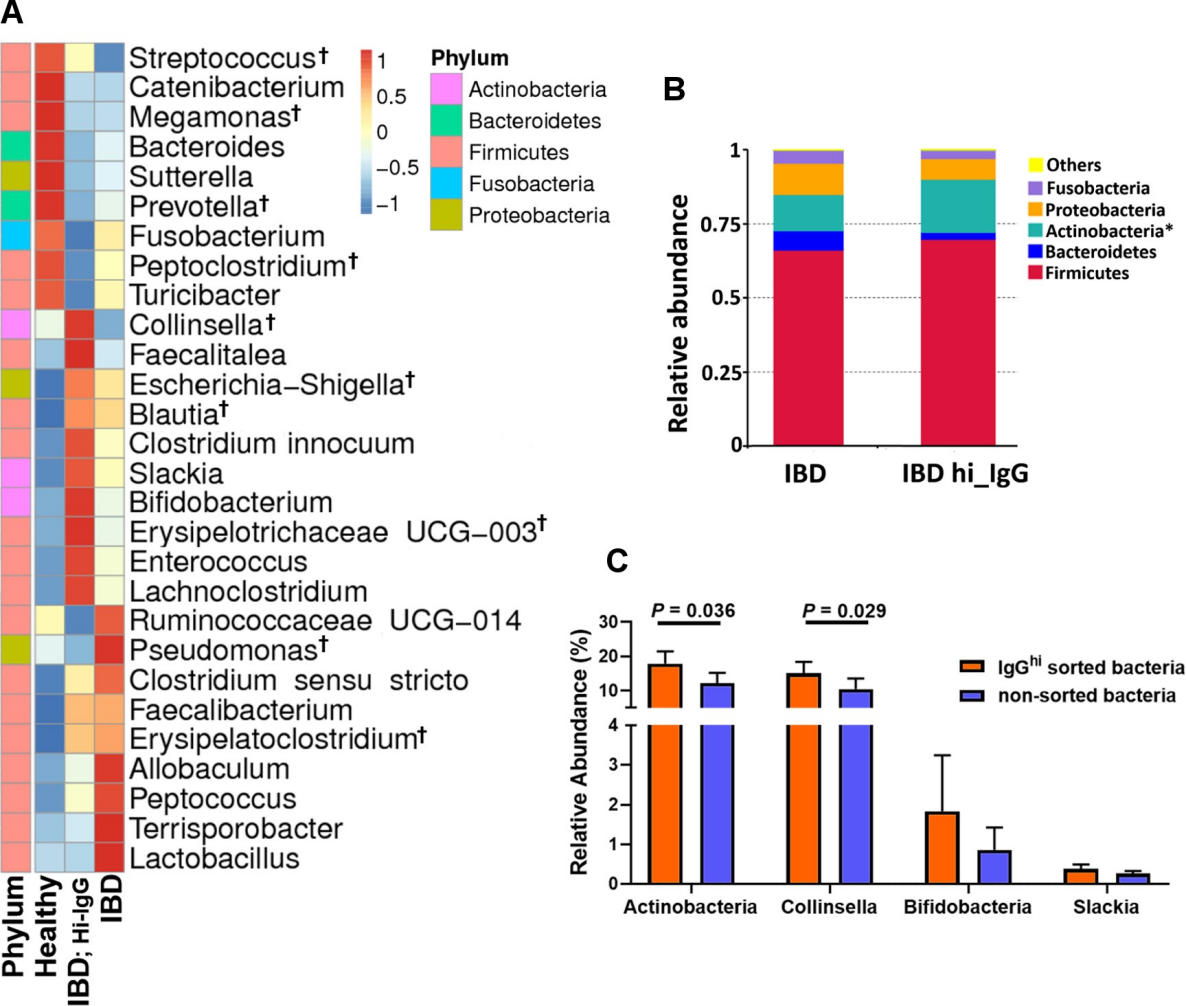
Microbiome analysis. IgG^hi^ sorted fecal bacteria from (n = 10) dogs with IBD, and non-sorted bacteria (n = 10; paired fecal samples from dogs with IBD) and bacteria from healthy control animals (n = 10) were analyzed by 16S rRNA sequencing, as described in Methods. (A) Species abundance heat map at taxonomic level representing average differences, with 0 = no difference, -1 and 1 representing maximum differences. (†) Showing the top 10 taxa abundance. (B) Bar graph depicting the relative abundance of 5 major phyla comparing the IgG^hi^ sorted population with non-sorted bacteria, obtained from same dogs with IBD. A significantly increased abundance of *Actinobacteria* phyla was found in IgG^hi^ sorted population. (C) Bar graph showed relative abundance comparing between IgG^hi^ sorted and non-sorted bacteria for members of *Actinobacteria* phyla. The data were reported as Mean ± SD, and statistical comparisons were calculated using paired t-test (*P ≤ 0.05, **P ≤ 0.01, ***P ≤ 0.001).

The overall composition and complexity of the GI microbiome in dogs with IBD (n = 10) was compared to that of healthy dogs (n = 10), using 16S rRNA metagenomics sequencing (Fig 6). Dogs with IBD had a greater abundance of bacteria in the *Proteobacteria* phylum (*P* = 0.045; S4 Fig) including *Escherichia-Shigella* and other genera such as *Clostridium*, *Blautia*, *Bifidobacterium*, *Enterococcus*, *Pseudomonas*, *Faecalibacterium*, *Lactobacillus*, along with a decrease in abundance of *Bacteroidetes* phyla (*P* = 0.048), and other genera; *Streptococcus*, *Fusobacterium*, *Peptoclostridium*, and *Turicibacter*, compared to the flora present in healthy control dogs (Fig 6A). These results are largely in agreement with previous studies of the microbiome in dogs with IBD and indicate that our study populations were similar to those of other studies with respect to bacterial diversity and differences in IBD versus healthy dog microbiomes [39–41].

We next conducted 16S sequencing studies to determine whether bacteria with high levels of IgG binding present in feces of dog with IBD represented uniquely enriched subsets of bacteria (e.g., potentially pathogenic bacteria), or whether the IgG^hi^ population of bacteria was evenly distributed amongst all the major phyla (i.e., no enrichment for specific phyla or genera). In part the rationale for this question was prior evidence for selective immunoglobulin binding to pathogenic bacteria in human patients with CD [21, 22].

Bacteria with the highest levels of IgG binding included *Collinsella*, *Faecalitalea*, *Escherichia-Shigella*, *Blautia*, *Bifidobacterium*, *Clostridium innocuum*, *Slackia* and *Enterococcus* (Fig 6A and S3 Table). The taxa with the lowest levels of IgG binding included *Pseudomonas*, *Clostridium* (sensu stricto) and *Lactobacillus*. We found that there was significant enrichment of bacteria in the *Actinobacteria* phylum (*P* = 0.036) in the IgG^hi^ population, compared to non-sorted IBD bacteria (Fig 6B and 6C and S3 Table**)**. Also, the most abundant genus in the IgG^hi^ *Actinobacteria* phylum was *Collinsella*, which was significantly enriched in the IgG^hi^ sorted population of bacteria compared to non-sorted bacteria. Thus, these studies indicated that there was preferential immune recognition of *Actinobacteria* in dogs with IBD.

### Sensitivity and specificity of fecal bacteria IgG assay for detection of IBD in dogs

**Fig 7:**
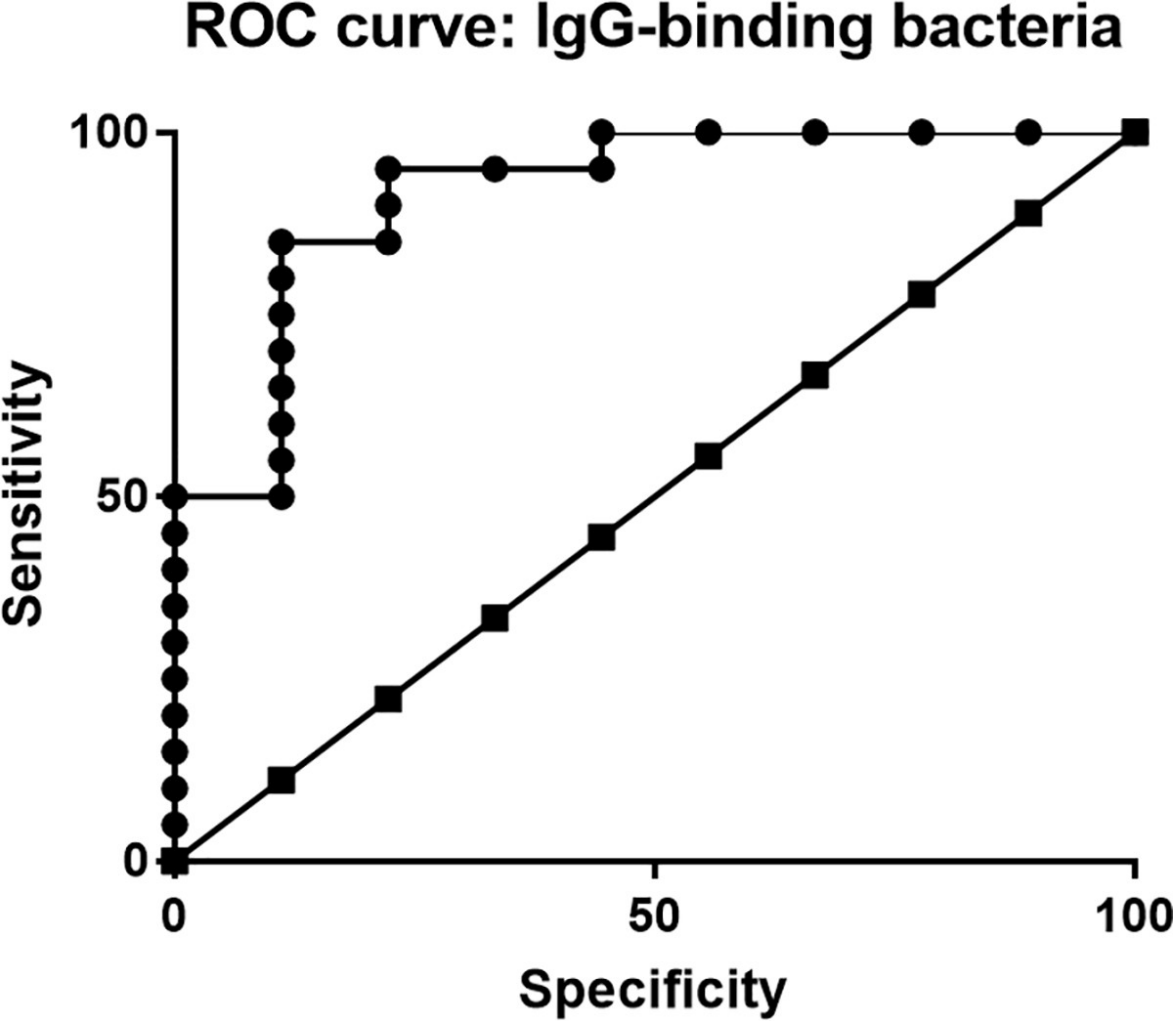
Receiver operator curves for bacterial IgG assay. To quantify the diagnostic ability of the bacterial IgG assay to discriminate dogs with IBD (n = 20) from normal dogs (n = 9) based on percentage IgG-binding gut bacteria, ROC curve analysis was performed. Area under the curve (AUC) was reported as 0.92, SD 0.06, *P* < 0.0001.

The preceding results suggested that quantitation of the relative degree of IgG binding to fecal bacteria might be useful as a diagnostic test for detection of IBD in dogs. Therefore, we evaluated the sensitivity and specificity of the flow cytometric assay, evaluating either percentage IgG^+^ bacteria, or amount of IgG bound per bacterial cell (i.e., MFI), for sensitivity and specificity for detecting IBD in dogs. Using receiver operating curves (ROC) (Fig 7), we found that the area under the curve (AUC) for IgG^+^ bacteria was 0.92 (95% CI: 0.80–1.03, *P* < 0.0001). This result indicated high diagnostic utility for the flow cytometric test using percentage of IgG^+^ bacteria for differentiating IBD dogs from healthy dogs.

Next, we evaluated the sensitivity and specificity of the bacterial IgG assay, using a cutoff point based on the upper limit of 95% confidence interval determined using bacteria from healthy dogs, which was defined as 60% IgG^+^ bacteria. The bacterial percentage IgG^+^ assay was found to have 85% sensitivity (95%CI: 62.11–96.79) and 89% specificity (95%CI: 51.75–99.72) for detection of clinically apparent IBD in dogs. Overall, the fecal IgG test in dogs had a positive likelihood ratio of 7.7 and a negative likelihood ratio of 0.17. However, the percentage of IgG^+^ bacteria was found to not correlate with the disease activity index, including CIBDAI (*P* = 0.71) and CCECAI (*P* = 0.55). In addition, the overall histopathologic score and endoscopic lesion scores did not correlate with the percentage of IgG^+^ bacteria, as determined by linear regression analysis. Thus, the assessment of IgG bound to the surface of bacteria was found to be a very sensitive and specific test for detection of IBD in dogs, though test positivity did not correlate with disease activity or severity. Thus, the fecal IgG assay was considered to have higher sensitivity and specificity for IBD detection than other currently available assays, including fecal calprotectin (S100A12), which was found to be 65% sensitive and 84% specific for diagnosing IBD in dogs [42].

## Discussion

The interaction between the host immune response and gut bacteria is now considered a primary driver of intestinal inflammation in humans with IBD [6–8]. For example, there is convincing evidence of an increase in IgG responses directed against gut bacteria in patients with Crohn’s disease [12, 18, 19]. For example, the presence of high levels of IgG^+^ bacteria was shown to be specific for Crohn’s disease; since high levels of IgG^+^ fecal bacteria were not detected in patients with infectious colitis [12, 18] or coeliac disease [43]. Similar studies have not been conducted previously in dogs with IBD and to the authors’ knowledge, this is the first study that documents an immune response against gut bacteria in this disease.

A key finding from our study was that dogs with IBD had a significantly higher binding of IgG to gut bacteria, compared to healthy dogs (Fig 1). For example, there were overall 30% more bacteria with surface bound IgG in dogs with IBD than in normal dogs (Fig 1A). These differences are roughly in agreement with results of bacterial IgG binding levels in previous studies in patients with CD and UC [12, 18]. Moreover, our studies also indicate that the source of the anti-bacterial IgG production was most likely from local immunoglobulin production in the gut, rather than from immunoglobulin produced in extra-intestinal sites, a result that was also noted in studies in patients with Crohn’s disease [7, 12]. However, we noted that a result of no difference in serum IgG recognizing *E*. *coli* isolates from IBD dogs and healthy could be truly due to lack of increased overall production of IgG-specific bacteria systemically or due to lack of IgG to *E*. *coli* in particular. So, we further evaluated IgG recognition of an *Enterococcus* fecal isolate, which is a Gram positive bacterium with high abundance in feces. We also found no difference of serum IgG response to this bacterium between IBD and healthy dogs (S5 Fig).

One difference between immunoglobulin responses to intestinal bacteria in our study and studies in Crohn’s patients is that we did not observe an increased in IgA binding to bacteria in dogs with IBD, whereas in humans there was significantly more IgA was present on gut bacteria compared to gut bacteria in healthy patients [12]. The reasons for this difference between species are not currently known, though it should be noted that soluble IgA concentrations have been shown to be lower overall in the feces of dogs with IBD than in healthy dogs [13, 44].

Our work also demonstrates a plausible link between bacterial IgG binding and induction of intestinal inflammation, which could involve activation of macrophages in the gut. For example, we found that incubation of macrophages with fecal bacteria from dogs with IBD triggered significantly greater macrophage activation and TNF-α production than did bacteria from healthy dog GI tracts (see Figs 4 and 5). Conversely, bacteria from IBD dogs triggered significantly less IL-10 production by macrophages than bacteria from healthy dogs. Thus, the net effect of the interaction of macrophages with IgG bound bacteria in animals with IBD would be to trigger greater local immune activation and inflammation. This effect could in part be mediated by the interaction of bacterial bound IgG with activating Fc receptors expressed by macrophages in the intestinal epithelium or lamina propria [44, 45]. We noted that our results showed more polarized Th1 response based on the direct effect on macrophage in vitro setting, which may not fully recapitulate the complexity of responses [46] occurring in the gut. Additionally, our studies assessed the phagocytic activity of tissue culture derived macrophages. However, it is possible that macrophages from the GI tract of dogs with IBD may have altered responses to these Ig-coating bacteria, exhibiting either impaired function or hyper-responsiveness compared to macrophages from the GI tract of healthy dogs. Thus, further study would be needed, for example to isolate mucosal macrophages from IBD dogs and compare their activity to gut macrophages from healthy dogs.

Interestingly, we also found that the IgG response in dogs with IBD appeared to be directed preferentially towards bacteria considered part of the dysbiotic flora present in the gut, as described in previous studies [24, 25, 39–41]. Thus, we found that bacteria in the genus *Collinsella* had the highest levels of IgG binding in dogs with IBD, whereas this organism was not present in greater abundance in the gut of healthy dogs (see Fig 6A and S3 Table). This genus was noted in the gut microbiome in dogs with gastric-dilation and volvulus [47] as well as reported to be one of the high IgA binding bacteria detected in patients with CD [11, 48]. While not all dysbiotic flora are pathogenic, certainly some of the genera represented in the dysbiotic gut (e.g., *Escherichia*, *Clostridium* and *Enterococcus*) have been associated with intestinal infection and invasion [49–52]. These pathogenic bacteria, particularly if enteroinvasive or capable of enhanced GI colonization, could trigger greater immune recognition and local antibody production [52–54]. Besides sequencing, future studies focusing on the spatial distribution of mucosal-associated microbiota including *Collinsella* spp. assessed by techniques such as fluorescence in situ hybridization would be useful.

In terms of diagnostic utility, our studies demonstrated that the use of a bacterial flow cytometric IgG assay provided significant sensitivity and specificity for differentiating dogs with IBD from healthy dogs, using fecal samples (Fig 7). Since currently there are no commercially available assays for accurately identifying dogs with IBD, this bacterial flow cytometric assay may provide a useful clinical test that can be run on fresh or frozen fecal samples.

Interestingly we observed no association between the level of IgG^+^ bacteria and clinical parameters associated with disease activity index, histopathology score, or endoscopic score. Like previous IBD studies in dogs, clinical parameters showed no correlation or a weak correlation with other IBD parameters including serum Ig, C-reactive protein and Calprotectin [13, 55, 56]. However, the presence of bacteria in the genus *Collinsella*; which we found to have the highest IgG binding in dogs with IBD (see Fig 6C); showed the highest association with common IBD clinical parameters including CIBDAI (*P* = 0.032), CCECAI (*P* = 0.024), histopathology scores (*P* = 0.016) and serum folate concentrations (*P* = 0.008, see S6 Fig) in agreement with previous studies in humans and in cats [48, 57]. In humans, *Collinsella* is considered as one of the taxa used to discriminate between patients with UC and CD [48].

Using a culture-independent approach of 16S rRNA metagenomic sequencing for microbiome analysis, our study demonstrated relatively good agreement with prior sequencing studies of IBD in dogs, analyzing either fecal or mucosa-associated microbiota [24, 25, 39–41]. For example, one prior study found dysbiosis of commensal bacteria, including increased *Proteobacteria* (e.g., *E*. *coli*), *Clostridium*, and *Enterococcus* [41]. In our study, these expanded populations of dysbiotic bacteria were found to have high levels of IgG bound on their surface (Fig 6A) resulting in increased overall percentage and higher overall MFI of IgG-binding bacteria in dogs with IBD (see Fig 1). However, some of the highest IgG binding taxa identified in our study were considered as non-IBD associated taxa in previous studies in dogs with IBD, including *Faecalibacterium*, *Allobaculum*, *Slackia* and *Clostridium* [40].

One potential limitation to our study was that some of the dogs with IBD had received antibiotic therapy prior to their enrollment in our study. Antibiotic treatment is known to significantly alter the intestinal microbiome in dogs [58, 59]. However, we found no significant difference in the overall percentages of IgG^+^ bacteria in dogs regardless of their antibiotic pre-treatment status (S7 Fig), suggesting that antibiotic treatment had a little discernable effect on generation of anti-bacterial IgG in dogs with IBD. This is an important observation because it suggests that the fecal IgG assay may be relatively resistant to potential interference by prior antimicrobial therapy.

In summary, our studies indicate that a high percentage of intestinal bacteria are recognized by IgG produced locally in the gut in dogs with IBD, and that IgG bound bacteria may be linked to intestinal inflammation. These results should be confirmed by larger studies in dogs with IBD and compared to animals with other causes of signs of GI dysfunction, such as viral infection, dietary changes, and infections with GI parasites (e.g., hookworm, whipworm, or Giardia infections). Such studies could provide additional insights into the role of local gut immune responses against gut bacteria in IBD and other GI inflammatory diseases.

## Conclusion

This study found that bacteria present in the gut of dogs with IBD had significantly higher levels of IgG binding than bacteria in the gut of healthy dogs. In addition, the highest level of IgG binding appeared to be directed against certain phyla of dysbiotic bacteria, with a significant great preference for *Actinobacteria*. In addition, IgG-coated bacteria from dogs with IBD triggered significantly greater macrophage TNF-α production than bacteria from healthy dogs, indicative of an inherently pro-inflammatory effect of increased IgG binding to bacteria. These findings suggest that humoral immune recognition of endogenous gut bacteria may be an important mediator of intestinal inflammation in dogs with IBD, and indicate relatedness in the immune pathogenesis of certain types of IBD in dogs and in man.

## Supporting information

S1 Table Demographic data, diet and histopathologic evaluation of IBD group. Table reported data from individual dog. WSAVA histopathologic score was reported from assessment of duodenal tissue biopsy.
(DOCX)

S2 Table Demographic data, and diet of normal group. (DOCX)

S3 Table IgG^hi^-sorted bacteria abundance. Table reported the comparison of % relative abundance between IgG^hi^-sorted and non-sorted bacteria from IBD group. Data shown in Mean ± SD (if parametric data) and Median (range) (if non-parametric data). The appropriate statistical comparison of 2 groups either paired t-test and Mann-Whitney test was performed corresponding the type of data. P value of 0.05 is set.
(DOCX)

S1 Fig Flow cytometry analysis and gating. Fecal bacteria were analyzed based on size and complexity corresponding to bacteria population as well as selective counting of 10^5^ bacteria cells. The percentage of positive fluorescence cells of IgG-binding bacteria and fluorescence intensity was analyzed by comparing to background threshold.
(TIF)

S2 Fig Association between IgG and IgA binding to fecal bacteria. Scatter dot plot of (A), percentage of IgA-bound and IgG-bound bacteria and (B) amount of IgG and IgA binding to individual bacteria (MFI) depicted. To analyze the degree of association between IgG and IgA binding, linear regression analysis was performed. The percentage of IgA-bound bacteria was significant correlated with the percentage of IgG-bound bacteria (R^2^ = 0.45, *P* = 0.001). Also, degree of IgA and IgG binding also showed significant correlation (R^2^ = 0.48, *P* = 0.001). Dashed lines depict 95% confidence band.
(TIF)

S3 Fig Serum IgA recognition of *E*. *coli* isolated from healthy dogs and dogs with IBD. Six separate fecal isolates of *E*. *coli* (3 from IBD and 3 from healthy dogs) were incubated with serum from dogs with IBD (n = 20) and healthy dogs (n = 9), and IgA binding to the surface of bacteria was quantitated using flow cytometry, as noted in Methods. Scatter plots depicting IgA^+^ bacteria percentages in healthy versus IBD dogs plotted. (†) Indicated the isolates from normal dog, while (‡) indicated the isolates from dog with IBD. Data were plotted as Mean ± SD.
(TIF)

S4 Fig Relative abundance of 5 major phyla in dogs with IBD and healthy controls. Significant decrease in *Bacteroidetes* (*P* = 0.048) and increased *Proteobacteria* (*P* = 0.045) were observed in dogs with IBD. Bar graphs depict relative abundance of 5 phyla, and statistical differences calculated using unpaired t-test (*P ≤ .05, **P ≤ .01, ***P ≤ .001).
(TIF)

S5 Fig Serum IgG recognition of *Enterococcus* isolated from healthy dogs and dogs with IBD. Two separate fecal isolates of *Enterococcus spp*. (1 from IBD and 1 from healthy dog) were incubated with serum from dogs with IBD (n = 20) and healthy dogs (n = 9), and IgG binding to the surface of bacteria was quantitated using flow cytometry, as noted in Methods. Scatter plots depicting IgG^+^ bacteria percentages in healthy versus IBD dogs plotted. (†) Indicated the isolate from normal dog, while (‡) indicated the isolate from dog with IBD. Data were plotted as Mean ± SD.
(TIF)

S6 Fig Association of *Collinsella* and clinical parameters in IBD. Scatter dot plot of % abundance of *Collinsella* and clinical parameters depicted. Linear regression analysis was performed. The P value as stated in the figures. Dashed lines depict 95% confidence band. CIBDAI; Canine Inflammatory Bowel Disease Activity Index, CCECAI; Canine Chronic Enteropathy Clinical Activity Index.
(TIF)

S7 Fig % IgG^+^ fecal bacteria comparison of IBD with or without antibiotic pretreatment. The percentages of IgG^+^ bacteria are plotted comparing IBD dogs with antibiotic pretreatment (n = 11) and no treatment (n = 9). Data are plotted as Mean ± SD. No statistical difference was found by unpaired t-test (*P* = 0.99).
(TIF)
