# Supplementary material for: Humoral immune responses against gut bacteria in dogs with inflammatory bowel disease
Source: PLoS One. 2019 Aug 1;14(8):e0220522. doi: 10.1371/journal.pone.0220522 (PMC6675102; doi:10.1371/journal.pone.0220522)
Supplement: S1 Table — Table reported data from individual dog. WSAVA histopathologic score was reported from assessment of duodenal tissue biopsy. (DOCX) [file pone.0220522.s001.docx]

**S1 Table. Demographic data, diet and histopathologic evaluation of IBD group**.

| No. | Breed | Age  (yr) | BCS  (/9) | CIBDAI | CCECAI | Diet | Histopathology | WSAVA  score |
| --- | --- | --- | --- | --- | --- | --- | --- | --- |
| 1  RG | Yorkshire Terrier | 3 | 4 | 10 | 14 | Commercial dog food; urinary care formula | 1. Mild lymphoplasmacytic enteritis with marked glandular degeneration.  2. Mild lymphoplasmacytic colitis. | 6 |
| 2  RC | Yorkshire Terrier | 2 | 4 | 10 | 13 | Commercial dog food with buffalo meat | Mild lymphoplasmacytic enteritis | 3 |
| 3  WT | Bernese Mountain Dog | 6 | 4 | 7 | 9 | Any food if dog eats | Mild lymphoplasmacytic and suppurative enteritis with some eosinophils and gland degeneration | 6.5 |
| 4  SF | Bernese Mountain Dog | 9 | 5 | 9 | 9 | Commercial dog food with whitefish and any food if dog eats | 1. Duodenum: Moderate lymphoplasmacytic and eosinophilic enteritis.  2. Ileum: Mild lymphoplasmacytic and eosinophilic enteritis.  3. Mild lymphoplasmacytic and eosinophilic colitis | 7 |
| 5  Mu | Bernese Mountain Dog | 6 | 5 | 2 | 3 | Commercial dog food with whitefish and potato | 1. Duodenitis, lymphoplasmacytic and eosinophilic, chronic–active, moderate with crypt abscesses, mild lacteal dilation and mild lymphoplasmacytic intraepithelial infiltration  2. Ileitis, lymphoplasmacytic and eosinophilic, chronic, moderate with mild lymphocytic intraepithelial infiltration  3. Colonic inflammation, lymphoplasmacytic and eosinophilic, chronic, mild, with mild lymphocytic intraepithelial infiltration | 5 |
| 6  Mi | Bernese Mountain Dog | 2 | 5 | 9 | 11 | Homemade diet with lamb and rice | 1.Moderate lymphoplasmacytic to eosinophilic duodenitis  2.Mild suppurative colitis with moderate numbers of globular leukocytes | 5 |
| 7  Ag | Mixed breed | 6 | 4 | 2 | 5 | Commercial dog food with whitefish and potato | 1. Duodenum and Ileum: Moderate eosinophilic and lymphoplasmacytic enteritis with villous shortening 2. Minimal lymphoplasmacytic colitis. | 6 |
| 8  BD | Mixed breed | 15 | 6 | 3 | 3 | Commercial dog food with rabbit, wild bison, grain-free | Mild lymphoplasmacytic and eosinophilic enteritis | 4 |
| 9  Ka | Mixed breed | 12 | 6 | 2 | 2 | Commercial dog food with novel protein | Moderate, chronic, regionally extensive lymphoplasmacytic enteritis | 1 |
| 10  Ov | Mixed breed | 2 | 4 | 3 | 4 | Commercial dog food; high energy for GI | Moderate lymphoplasmacytic enteritis. | 5 |
| 11  C | Labrador Retriever | 12 | 4 | 9 | 10 | Commercial canned dog food with buffalo and salmon | Mild lymphoplasmacytic and eosinophilic enteritis | 2 |
| 12  JR | Labrador Retriever | 5 | 1 | 7 | 8 | High caloric commercial puppy food | 1. Duodenum: Enteritis, lymphoplasmacytic, chronic, moderate with villus fusion.  2. Ileitis, lymphoplasmacytic and eosinophilic, chronic, severe with villus fusion  3. Colitis, lymphoplasmacytic, chronic, mild with lamina propria fibrosis. | 5 |
| 13  LS | Pug | 10 | 3 | 11 | 13 | Homemade diet; chicken, rice and sweet potato | 1. Severe catarrhal enteritis in duodenum and ileum, with bacterial colonies on the surface  2. Duodenum, Ileum: increased lymphocytes and plasma cells throughout the lamina propria of the mucosa | 12 |
| 14  R | Rottweiler | 6 | 4 | 14 | 19 | Commercial dog food; weight management formula | 1. Duodenum: Severe, interstitial, lymphoplasmacytic and eosinophilic enteritis, with mild villus blunting, mild lacteal dilation, and formation of crypt abscesses  2. Ileum: Mild to moderate, interstitial, lymphoplasmacytic and eosinophilic enteritis, with mild to moderate lacteal dilation  3. Colon: Mild, patchy lymphoplasmacytic colitis | 10.5 |
| 15  Hk | Boxer | 4 | 5 | 4 | 4 | Commercial dog food with novel protein | Severe lymphoplasmacytic and eosinophilic enteritis | 2 |
| 16  CG | Cavalier King Charles Spaniel | 3 | 5 | 2 | 2 | Commercial dog food with turkey and potato | 1. Mild to moderate lymphoplasmacytic enteritis with glandular degeneration and villous blunting.  2. Mild lymphoplasmacytic colitis. | 6 |
| 17  Ok | German Shorthaired Pointer | 4 | 4 | 5 | 6 | Commercial dog food; low fat for GI | Duodenum/Ileum/Colon: Chronic moderate lymphoplasmacytic and mild eosinophilic enterocolitis, few crypt abscesses, and mild lymphangiectasia. | 6 |
| 18  AJ | English  Bulldog | 7 | 4 | 7 | 8 | Commercial dog food with duck, potato | 1. Duodenum: Moderate lymphoplasmacytic enteritis with few neutrophils and moderate to marked intraepithelial lymphocytes infiltration.  2. Ileum: Minimal lymphoplasmacytic enteritis with mild intraepithelial lymphocyte infiltration.  3. Colon: Minimal lymphoplasmacytic enteritis. | 5 |
| 19  HL | American Eskimo | 10 | 3 | 9 | 10 | Homemade diet with cooked chicken | 1. Duodenum: Moderate lymphoplasmacytic duodenitis  2. Ileum: minimal presence of lymphocytes and plasma cells within the lamina propria  3. Colon: Mild lymphoplasmacytic and eosinophilic colitis | 3 |
|  |  |  |  |  |  |  |  |  |
| 20  Ko | Siberian Husky | 4 | 3 | 8 | 9 | Commercial dog food with hydrolyzed protein | 1. Duodenum: Moderate, diffuse, chronic, lymphoplasmacytic enteritis with moderate villous blunting  2. Ileum: Mild, diffuse, chronic, lymphoplasmacytic ileitis  3. Colon: Mild, diffuse, chronic, lymphoplasmacytic colitis | 3 |
|  |  |  |  |  |  |  |  |  |
