# Supplementary material for: Humoral immune responses against gut bacteria in dogs with inflammatory bowel disease
Source: PLoS One. 2019 Aug 1;14(8):e0220522. doi: 10.1371/journal.pone.0220522 (PMC6675102; doi:10.1371/journal.pone.0220522)
Supplement: S2 Table — (DOCX) [file pone.0220522.s002.docx]

**S2 Table. Demographic data, and diet of normal group**.

| No. | Breed | Age  (yr) | BCS  (/9) | Diet |
| --- | --- | --- | --- | --- |
| 1  Dm | English Setter | 9 | 5 | Commercial dog food with venison |
| 2  Mm | English Coonhound | 12 | 7 | Commercial dog food; weight control formula |
| 3  J | Mixed breed | 6 | 5 | Commercial dog food; standard formula |
| 4  LL | Mixed breed | 9 | 6 | Commercial dog food; senior formula |
| 5  Rx | Cocker Spaniel | 5 | 5 | Commercial dog food; standard formula |
| 6  Ch | Standard Poodle | 6 | 5 | Commercial dog food; standard formula |
| 7  MH | Nova Scotia Duck Tolling Retriever | 5 | 5 | Commercial dog food with lamb and rice |
| 8  T | Chihuahua | 12 | 4 | Commercial canned dog food with venison |
| 9  Td | Shi Tsu | 4 | 6 | Commercial dog food; standard formula |
|  |  |  |  |  |
