# Supplementary material for: Humoral immune responses against gut bacteria in dogs with inflammatory bowel disease
Source: PLoS One. 2019 Aug 1;14(8):e0220522. doi: 10.1371/journal.pone.0220522 (PMC6675102; doi:10.1371/journal.pone.0220522)
Supplement: S3 Table — Table reported the comparison of % relative abundance between IgGhi-sorted and non-sorted bacteria from IBD group. Data shown in Mean ± SD (if parametric data) and Median (range) (if non-parametric data). The appropriate statistical comparison of 2 groups either paired t-test and Mann-Whitney test was performed corresponding the type of data. P value of 0.05 is set. (DOCX) [file pone.0220522.s003.docx]

**S3 Table.** **IgG^hi^-sorted bacteria abundance**.

| Taxa | IBD | | *P* value |
| --- | --- | --- | --- |
|  | **IgG^hi^ bacteria (n=10)** | **Non-sorted bacteria**  **(n=10)** |  |
| *Actinobacteria*  *Bifidobacteria*  *Slackia*  *Collinsella* | 17.95 ± 11.08  0.3 (14.4)  0.38 ± 0.36  15.1 ± 10.66 | 12.23 ± 9.363  0.25 (5.8)  0.26 ± 0.21  10.5 ± 9.75 | **0.036**  0.094  0.058  **0.029** |
| *Bacteroidetes*  *Bacteroides*  *Prevotella* | 2.38 ± 1.82  1.08 ± 0.77  0.34 (2.74) | 6.64 ± 4.07  2.43 ± 2.53  1.19 (7.56) | 0.071  0.572  0.131 |
| *Firmicutes*  *Lachnoclostridium*  *Megamonas*  *Faecalitalia*  *Catenibacterium*  *Clostridium sensu stricto*  *Blautia*  *Enterococcus*  *Streptococcus*  *Clostridium innoculum*  *Lactobacillus*  *Erysipelotrichaceae*  *Turicibacter*  *Peptoclostridium*  *Erysipelatoclostridium*  *Faecalibacterium* | 69.64 ± 10.7  0.3 (0.3)  0.05 (0.78)  0.3 (0.6)  0.6 (3.8)  0.65 (2.8)  10.93 (27.03)  1.4 (12.2)  1.17 (19.69)  0.15 (0.8)  0.9 (4.7)  0.14 (18.8)  0 (0.1)  8.0 ± 4.19  1.96 (20.78)  0.8 (31.91) | 65.99 ± 12.85  0.2 (0.2)  0.06 (1.05)  0.2 (0.4)  0.75 (7.8)  0.7 (6.5)  9.65 (21.52)  0.8 (4.8)  0.75 (13.74)  0.1 (0.5)  1.35 (15.1)  0.45 (8.43)  0.1 (0.4)  10.26 ± 6.78  1.63 (26.47)  0.7 (31.87) | 0.156  0.176  0.77  0.093  0.477  0.3  0.492  0.089  0.275  0.140  0.348  **0.002**  **0.008**  0.313  0.921  0.766 |
| *Fusobacteria*  *Fusobacterium* | 2.77 ± 2.33  2.68 ± 2.31 | 4.41 ± 3.03  4.25± 2.94 | 0.215  0.223 |
| *Proteobacteria*  *Escherichia-Shigella*  *Sutterella*  *Pseudomonas* | 7.04 ± 6.81  2.67 (21.75)  0.05 (0.3)  0.05 (3.9) | 10.61 ± 9.9  1.57 (13.76)  0.1 (0.2)  1.18 (31.61) | 0.267  0.557  0.766  0.084 |

Table reported the comparison of % relative abundance between IgG^hi^-sorted and non-sorted bacteria from IBD group. Data shown in Mean ± SD (if parametric data) and Median (range) (if non-parametric data). The appropriate statistical comparison of 2 groups either paired t-test and Mann-Whitney test was performed corresponding the type of data. P value of 0.05 was set.
