# Supplementary figures and images for: Humoral immune responses against gut bacteria in dogs with inflammatory bowel disease
Source: PLoS One. 2019 Aug 1;14(8):e0220522. doi: 10.1371/journal.pone.0220522 (PMC6675102; doi:10.1371/journal.pone.0220522)

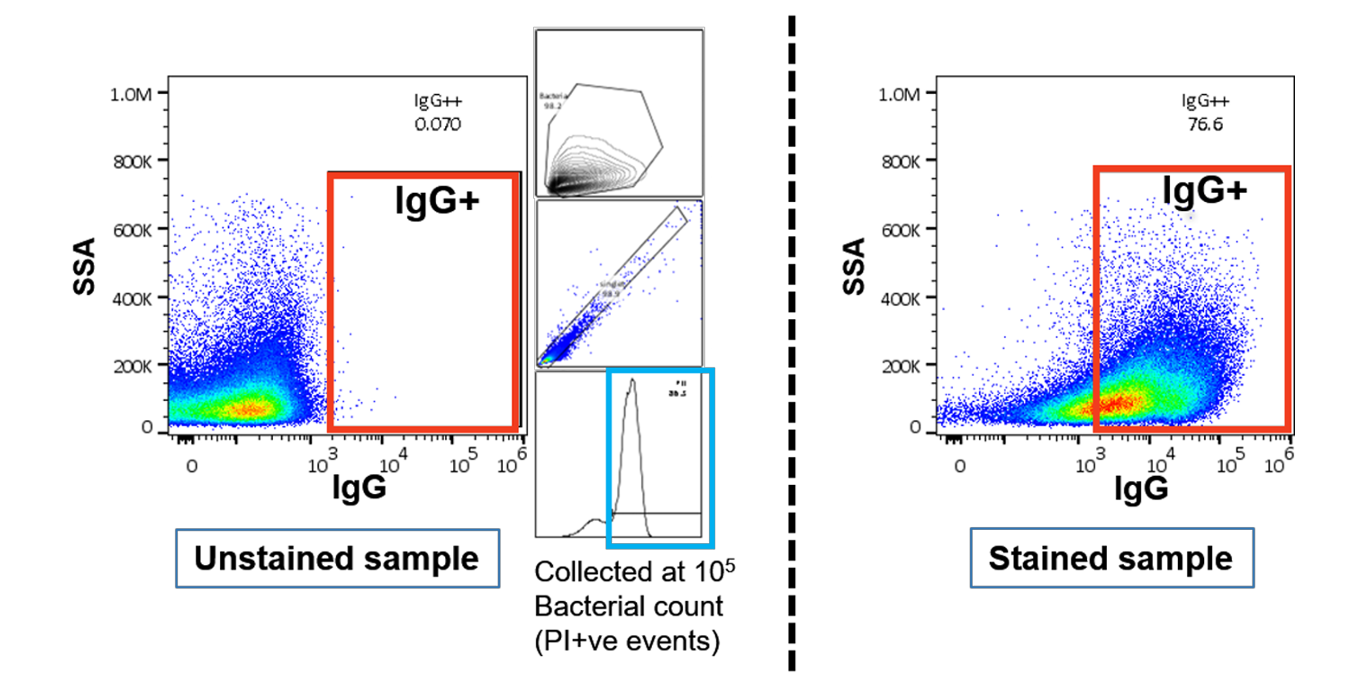

Supplement: S1 Fig — Fecal bacteria were analyzed based on size and complexity corresponding to bacteria population as well as selective counting of 105 bacteria cells. The percentage of positive fluorescence cells of IgG-binding bacteria and fluorescence intensity was analyzed by comparing to background threshold. (TIF) [file pone.0220522.s004.tif]

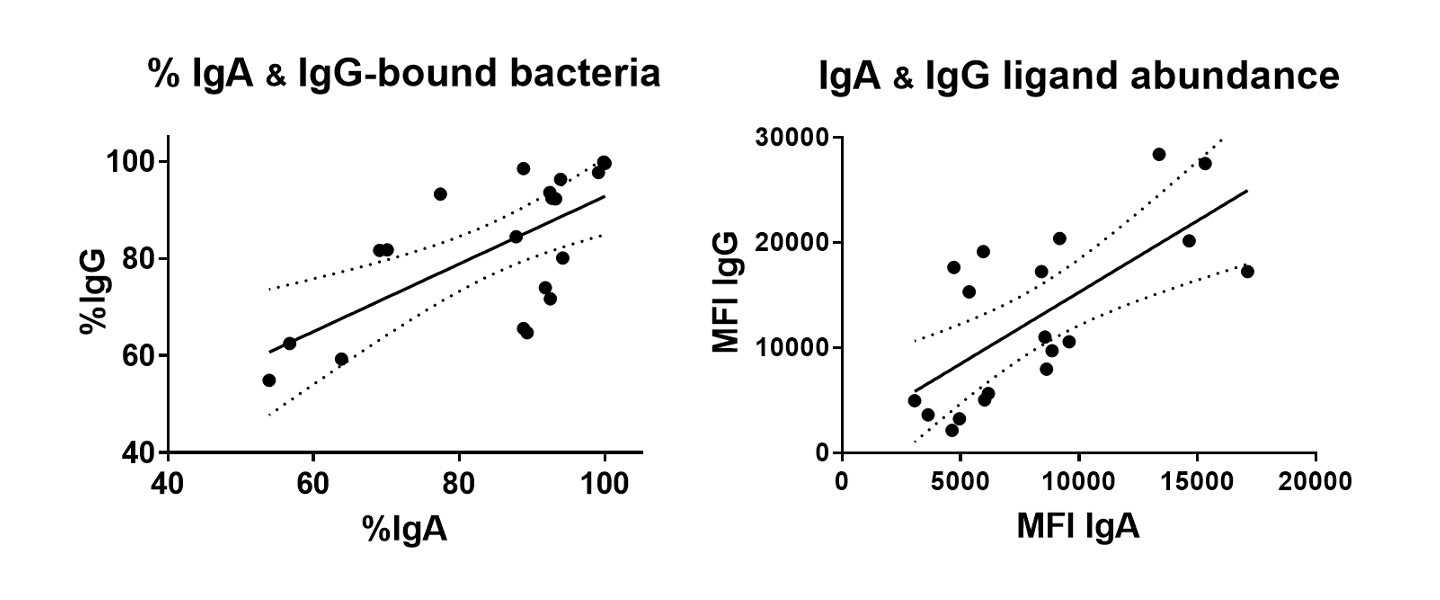

Supplement: S2 Fig — Scatter dot plot of (A), percentage of IgA-bound and IgG-bound bacteria and (B) amount of IgG and IgA binding to individual bacteria (MFI) depicted. To analyze the degree of association between IgG and IgA binding, linear regression analysis was performed. The percentage of IgA-bound bacteria was significant correlated with the percentage of IgG-bound bacteria (R2 = 0.45, P = 0.001). Also, degree of IgA and IgG binding also showed significant correlation (R2 = 0.48, P = 0.001). Dashed lines depict 95% confidence band. (TIF) [file pone.0220522.s005.tif]

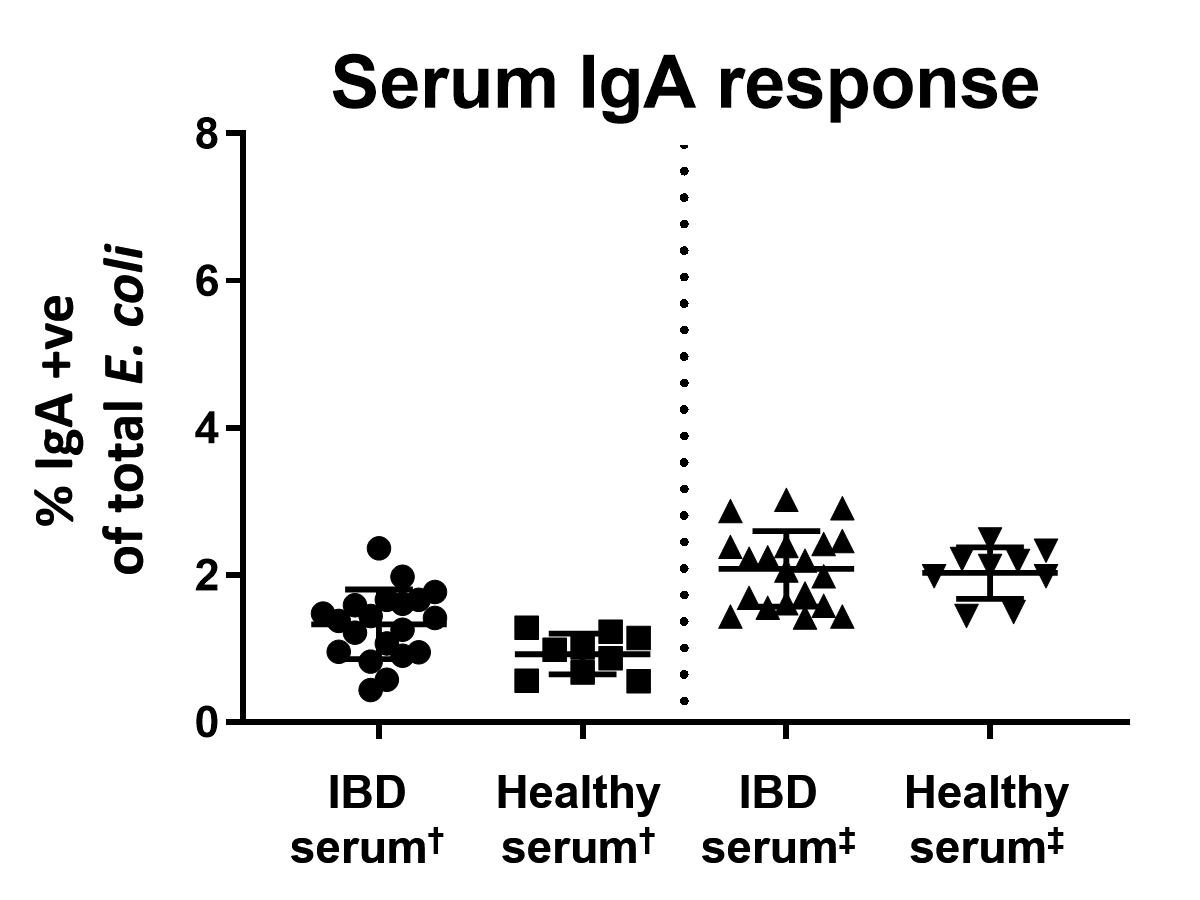

Supplement: S3 Fig — Six separate fecal isolates of E. coli (3 from IBD and 3 from healthy dogs) were incubated with serum from dogs with IBD (n = 20) and healthy dogs (n = 9), and IgA binding to the surface of bacteria was quantitated using flow cytometry, as noted in Methods. Scatter plots depicting IgA+ bacteria percentages in healthy versus IBD dogs plotted. (†) Indicated the isolates from normal dog, while (‡) indicated the isolates from dog with IBD. Data were plotted as Mean ± SD. (TIF) [file pone.0220522.s006.tif]

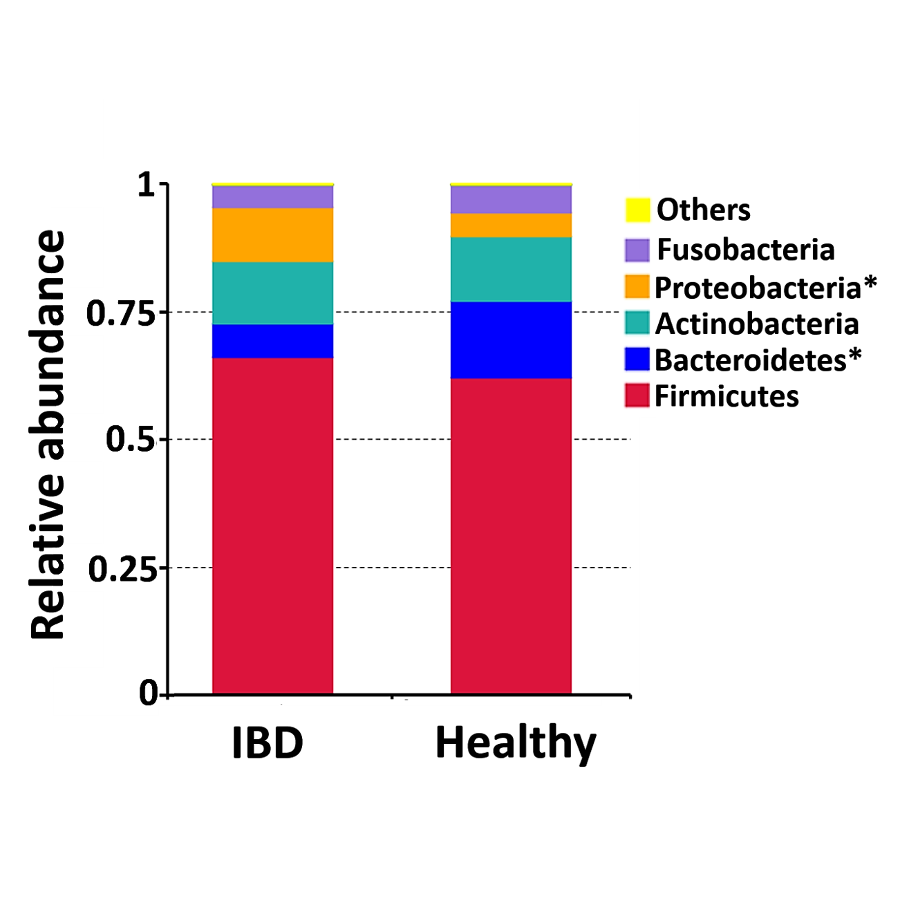

Supplement: S4 Fig — Significant decrease in Bacteroidetes (P = 0.048) and increased Proteobacteria (P = 0.045) were observed in dogs with IBD. Bar graphs depict relative abundance of 5 phyla, and statistical differences calculated using unpaired t-test (*P ≤ .05, **P ≤ .01, ***P ≤ .001). (TIF) [file pone.0220522.s007.tif]

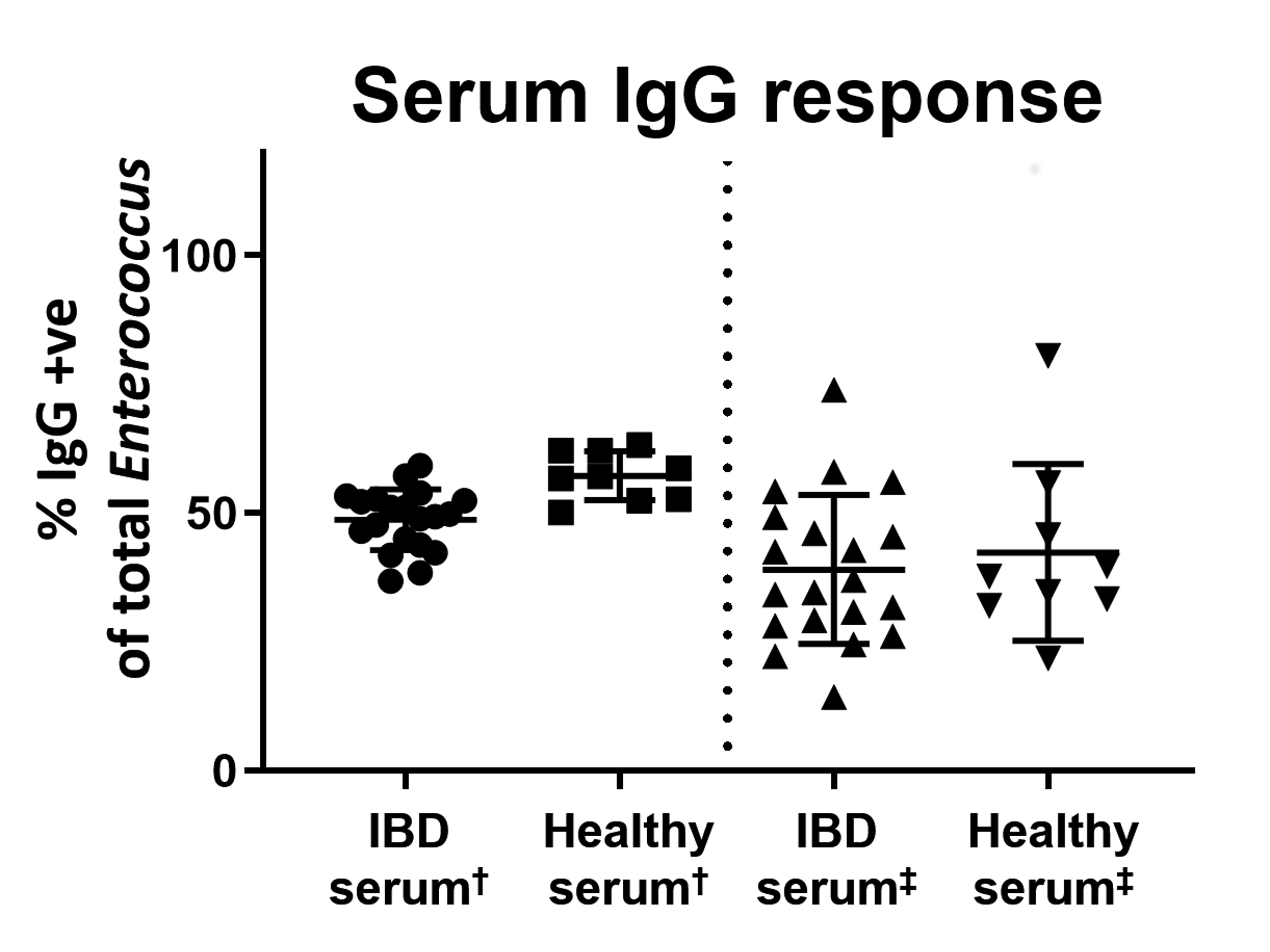

Supplement: S5 Fig — Two separate fecal isolates of Enterococcus spp. (1 from IBD and 1 from healthy dog) were incubated with serum from dogs with IBD (n = 20) and healthy dogs (n = 9), and IgG binding to the surface of bacteria was quantitated using flow cytometry, as noted in Methods. Scatter plots depicting IgG+ bacteria percentages in healthy versus IBD dogs plotted. (†) Indicated the isolate from normal dog, while (‡) indicated the isolate from dog with IBD. Data were plotted as Mean ± SD. (TIF) [file pone.0220522.s008.tif]

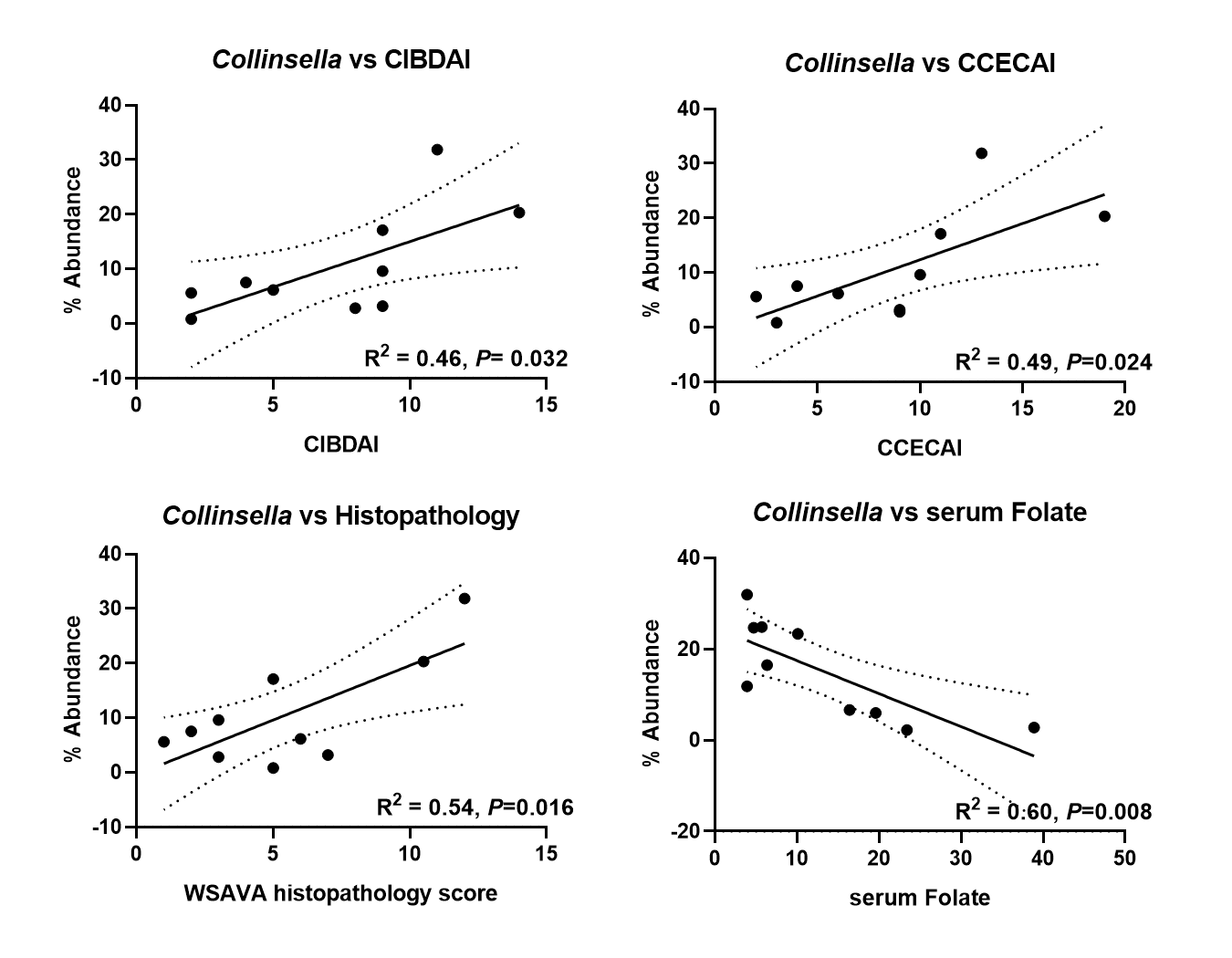

Supplement: S6 Fig — Scatter dot plot of % abundance of Collinsella and clinical parameters depicted. Linear regression analysis was performed. The P value as stated in the figures. Dashed lines depict 95% confidence band. CIBDAI; Canine Inflammatory Bowel Disease Activity Index, CCECAI; Canine Chronic Enteropathy Clinical Activity Index. (TIF) [file pone.0220522.s009.tif]

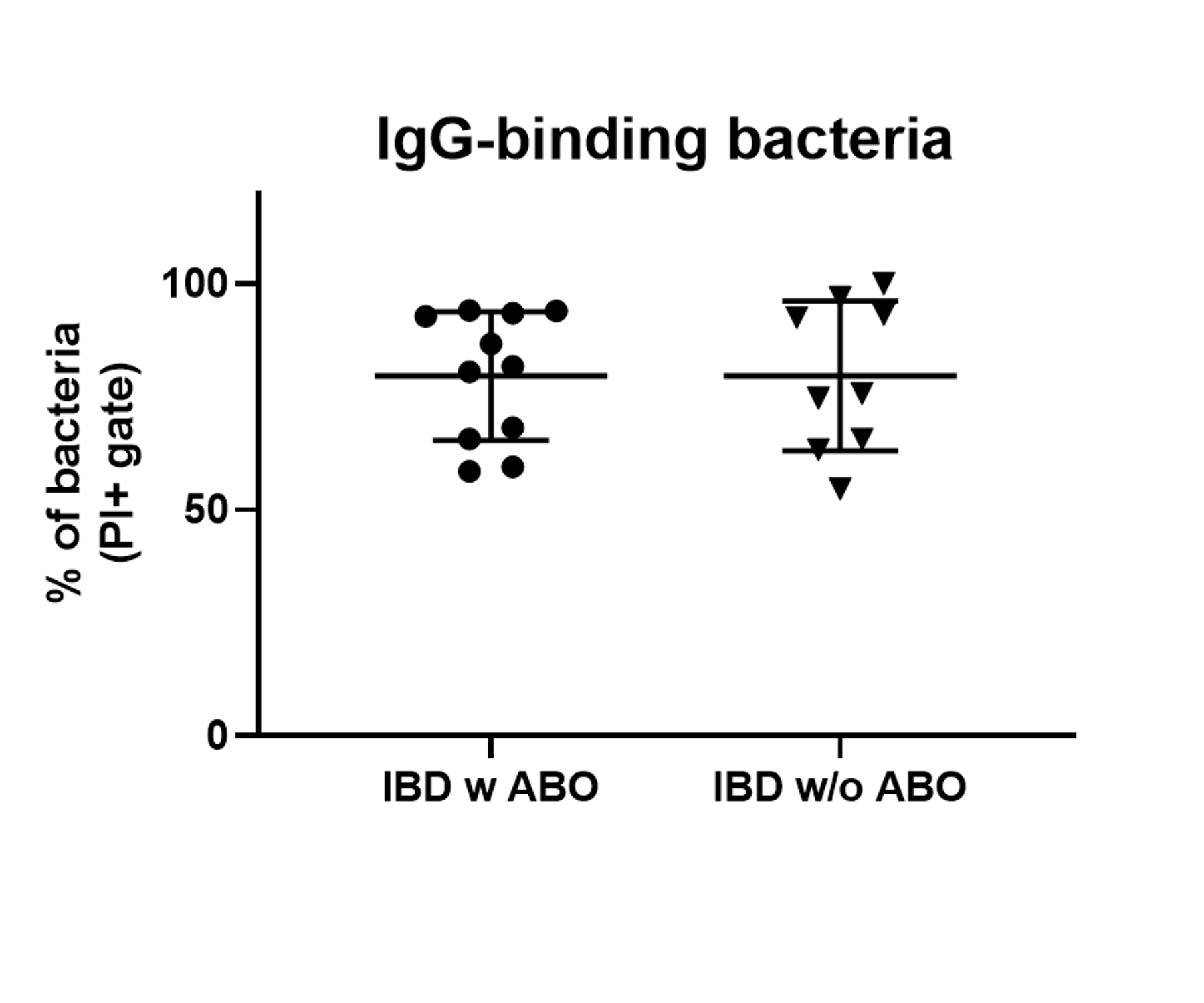

Supplement: S7 Fig — The percentages of IgG+ bacteria are plotted comparing IBD dogs with antibiotic pretreatment (n = 11) and no treatment (n = 9). Data are plotted as Mean ± SD. No statistical difference was found by unpaired t-test (P = 0.99). (TIF) [file pone.0220522.s010.tif]
